# 2-(4-Methylsulfonylphenyl)pyrimidines as Prospective Radioligands for Imaging Cyclooxygenase-2 with PET—Synthesis, Triage, and Radiolabeling

**DOI:** 10.3390/molecules23112850

**Published:** 2018-11-02

**Authors:** Michelle Y. Cortes-Salva, Stal Shrestha, Prachi Singh, Cheryl L. Morse, Kimberly J. Jenko, Jose A. Montero Santamaria, Sami S. Zoghbi, Robert B. Innis, Victor W. Pike

**Affiliations:** Molecular Imaging Branch, National Institute of Mental Health, National Institutes of Health, 10 Center Drive, Bethesda, MD 20892, USA; michelle.cortes@nih.gov (M.Y.C.-S.); stal.shrestha@med.miami.edu (S.S.); prachi1singh1@gmail.com (P.S.); morsec@mail.nih.gov (C.L.M.); kimjojenko@gmail.com (K.J.J.); jose.monterosantamaria@nih.gov (J.A.M.S.); zoghbis@mail.nih.gov (S.S.Z.) innisr@mail.nih.gov (R.B.I.)

**Keywords:** COX-2, radioligand, carbon-11, fluorine-18

## Abstract

Cyclooxygenase 2 (COX-2) is an inducible enzyme responsible for the conversion of arachidonic acid into the prostaglandins, PGG2 and PGH2. Expression of this enzyme increases in inflammation. Therefore, the development of probes for imaging COX-2 with positron emission tomography (PET) has gained interest because they could be useful for the study of inflammation in vivo, and for aiding anti-inflammatory drug development targeting COX-2. Nonetheless, effective PET radioligands are still lacking. We synthesized eleven COX-2 inhibitors based on a 2(4-methylsulfonylphenyl)pyrimidine core from which we selected three as prospective PET radioligands based on desirable factors, such as high inhibitory potency for COX-2, very low inhibitory potency for COX-1, moderate lipophilicity, and amenability to labeling with a positron-emitter. These inhibitors, namely 6-methoxy-2-(4-(methylsulfonyl)phenyl-*N*-(thiophen-2ylmethyl)pyrimidin-4-amine (**17**), the 6-fluoromethyl analogue (**20**), and the 6-(2-fluoroethoxy) analogue (**27**), were labeled in useful yields and with high molar activities by treating the 6-hydroxy analogue (**26**) with [^11^C]iodomethane, [^18^F]2-fluorobromoethane, and [*d*_2_-^18^F]fluorobromomethane, respectively. **[^11^C]17**, **[^18^F]20**, and **[*d*_2_-^18^F]27** were readily purified with HPLC and formulated for intravenous injection. These methods allow these radioligands to be produced for comparative evaluation as PET radioligands for measuring COX-2 in healthy rhesus monkey and for assessing their abilities to detect inflammation.

## 1. Introduction

Cyclooxygenase has two isoforms, COX-1 and COX-2, that exist in endoplasmic reticulum and produce prostaglandins G2 and H2 from arachidonic acid. Prostaglandins are important mediators of symptoms of inflammation, such as fever and pain [1]. COX-1 has been regarded as a widespread constitutive enzyme that is responsible for homeostatic prostaglandin synthesis. By contrast, COX-2 has been regarded as an inducible enzyme whose overexpression is implicated in the progression of many pathological and neurological disorders [2,3,4], including cancers [5], rheumatoid arthritis [6], cerebral ischemia [7], Alzheimer’s disease [2,3,4,8], and Parkinson’s disease [2,3,4,9]. Consequently, COX-2 could prove to be useful as a biomarker [1] for the early detection of inflammation in periphery and brain with positron emission tomography (PET), provided that effective PET radioligands can be developed.

Human COX-1 and COX-2 are 60% structurally similar with 70% amino acid homology and with very similar active site architecture [10]. For this reason, early searches for potent COX-2-selective inhibitors faced a considerable challenge [11]. Importantly, however, COX-2 has a hydrophobic pocket at its active site that is absent in COX-1 [12,13,14]. Ligand binding interactions within this pocket have been exploited to confer COX-2 inhibition selectivity. From these efforts, the COXIB’s have emerged as potent and COX-2 selective inhibitors that have served as a new class of nonsteroidal anti-inflammatory drug **(**NSAID). These drugs include celecoxib (Celebrex) [15], rofecoxib (Vioxx) [16], and valdecoxib (Bextra) [17].

**Chart 1:**
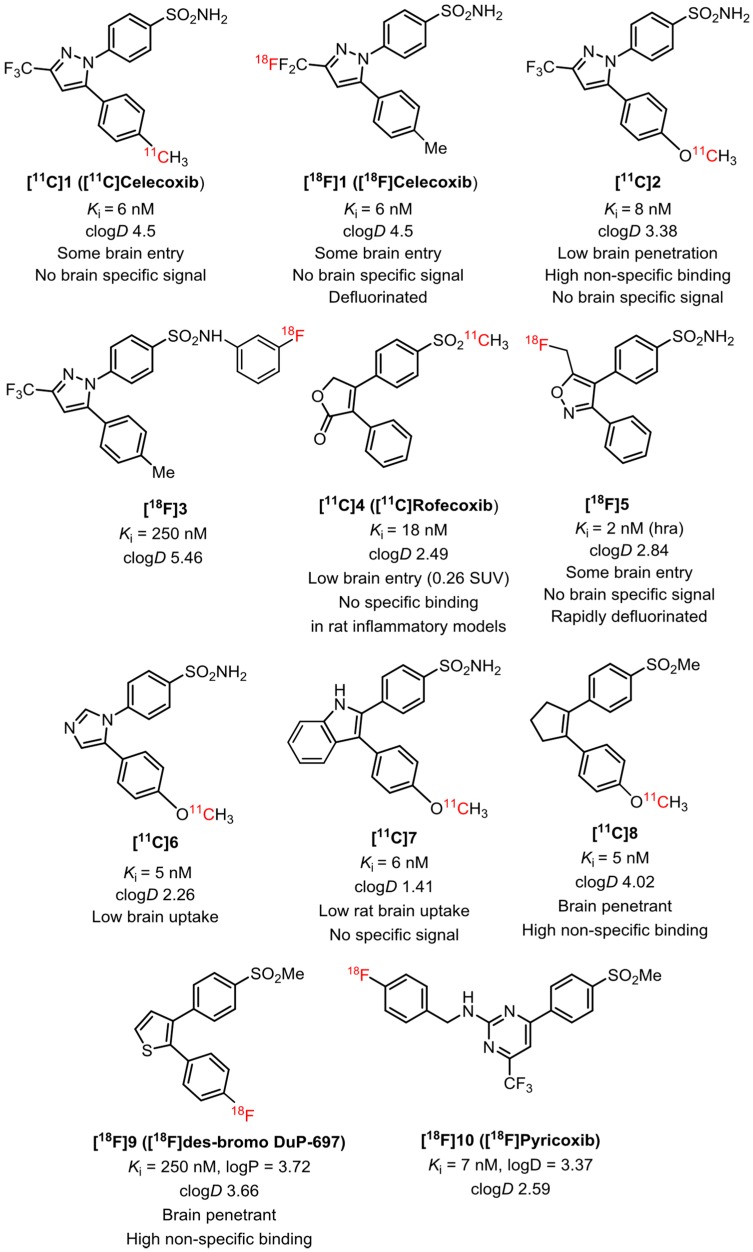
Some features of former candidate COX-2 positron emission tomography (PET) radioligands. Data are from the literature cited in the text, except for clog*D* values which were computed from 2-dimensional structure with Pallas software (see Experimental).

Numerous COX-2 inhibitors have been labeled with a positron-emitter, either carbon-11 (*t*_1/2_ = 20.4 min) or fluorine-18 (*t*_1/2_ = 109.8 min), and tested for imaging COX-2 in vivo with PET. Examples include [^11^C]celecoxib (**[^11^C]1**) [18,19], [^18^F]celecoxib (**[^18^F]1**) [20], and related compounds, such as **[^11^C]2** [21] and **[^18^F]3** [22], [^11^C]rofecoxib (**[^11^C]4**) [19,23,24], a radiofluorinated valdecoxib analog (**[^18^F]5**) [25], a diarylimidazole (**[^11^C]6**) [26], a diarylindole (**[^11^C]7**) [26], a diarylcyclopentene (**[^11^C]8**) [27], and [^18^F]desbromo-DuP-697 (**[^18^F]9**) [28,29]. All these compounds possess a pentacyclic core. Exceptionally, [^18^F]pyricoxib (**[^18^F]10**) [30] has a heterohexacyclic core (Chart 1). **[^18^F]10** has shown some promise for the detection of colorectal cancer [30] but no other effective radioligand has yet emerged for imaging COX-2 [31].

Successful PET radioligands for imaging proteins in living subjects must meet many requirements, among which high affinity and selectivity for the target protein, and moderate lipophilicity are the most critical [32]. For imaging in the brain, ability to penetrate the blood–brain barrier is further critical [33]. These requirements have not been well satisfied in most previous candidate COX-2 PET radioligands. Especially, few previous candidate radioligands have shown the ability to traverse the blood–brain barrier. For example, several contain an acidic primary or secondary arylsulfonamido group with a p*K*a expected to be in the range 7 to 9. These groups contribute strongly to high-affinity COX-2 binding but their high degree of ionization at physiological p*H* (7.4) counters brain entry. Nearly all the previously tested radioligands show COX-2 inhibitory potencies with *IC*_50_’s exceeding 5 nM whereas much higher affinity (indexed by 1/*IC*_50_) may be required for successful PET imaging.

We aim to develop a PET radioligand for imaging and quantifying human COX-2 under inflammatory conditions, as a tool for biomedical research and drug development targeting COX-2 [34]. In selecting leads for this purpose, we noticed 2-(4-methylsulfonylphenyl)pyrimidines as a class of compounds that contains some of the most potent and selective COX-2 inhibitors yet known [35], but which has not been investigated for leads to candidate PET radioligands. The structures of these compounds resemble those in the [^18^F]pyricoxib class in being based on a substituted pyrimidinyl ring (Chart 1), except that the ring substitution pattern is altered. Thus, the trifluoro group is replaced with another non-aryl group and the positioning of the aryl and substituted amino groups is reversed.

We prepared a set of compounds from the 2-(4-methylsulfonylphenyl)pyrimidine class, some already known, in a search for leads to PET radioligands that might fulfill requirements for imaging COX-2. We intended to use rhesus monkey as a preclinical model for radioligand evaluation. Therefore, we sought compounds with very high potency for inhibiting both monkey and human blood COX-2, and very high selectivity (>100-fold) for inhibiting COX-2 over monkey and human COX-1 based on enzymatic immunoassays. We also sought inhibitors that would show lipophilicity and other properties compatible with ability to traverse the blood–brain barrier (log*D* 2–3). From our set of prepared 2-(4-methylsulfonylphenyl)pyrimidines, we ultimately selected and produced three candidate PET radioligands, **[^11^C]17**, **[^18^F]20**, and **[*d*_2_-^18^F]27**. We concluded that these radioligands merit further investigation in non-human primate neuroinflammation and peripheral inflammation models.

## 2. Results and Discussion

### 2.1. Target Inhibitor Selection for Radioligand Development

We identified five 2-(4-methylsulfonylphenyl)pyrimidines (**17**–**19**, **22**, and **25**) from the report of Orjales et al. [35] that presented: (i) high potency for inhibition of human COX-2 from human whole blood (*IC*_50_ ≤ 5.1 nM); (ii) very low potency for inhibition of the isoform COX-1 from human whole blood (*IC*_50_ > 100 nM); (iii) computed lipophilicity in an acceptable range for a PET radioligand (clog*D* 2–3, ideally about 2.2); and (iv) at least one site that was expected to be amenable to labeling with either carbon-11 or fluorine-18. In addition to moderate lipophilicity, we sought other properties in these inhibitors that would be consistent with ability to traverse the blood–brain barrier, such as molecular weight of less than 500 Da, computed total polar surface area (tPSA) of less than 90 Å^2^, low hydrogen bond donor number (HBD), less than 9 heteroatoms, p*K*a less than 9.5, and absence of acidic ionizable groups such as sulfonamido and carboxyl groups [32] (Table 1). We designed another five previously unknown 2-(4-methylsulfonylphenyl)pyrimidines (**20**, **21**, **27**, **29**, and **30**) as potentially new and potent COX-2 inhibitors with other ostensibly favorable properties for PET radioligand development (Table 1). Each of these compounds carried a group that was expected to be readily amenable for labeling with either carbon-11 or fluorine-18 (i.e., in Table 1 the R^1^ group with carbon-11 for **21** and with fluorine-18 for **20**, **27**, and **29**; and the R^3^ group with carbon-11 for **30**). We also prepared two hydroxy compounds with low COX inhibitory potency [35] as possible labeling precursors, namely **24** and **26**.

### 2.2. Chemistry

**Scheme 1:**
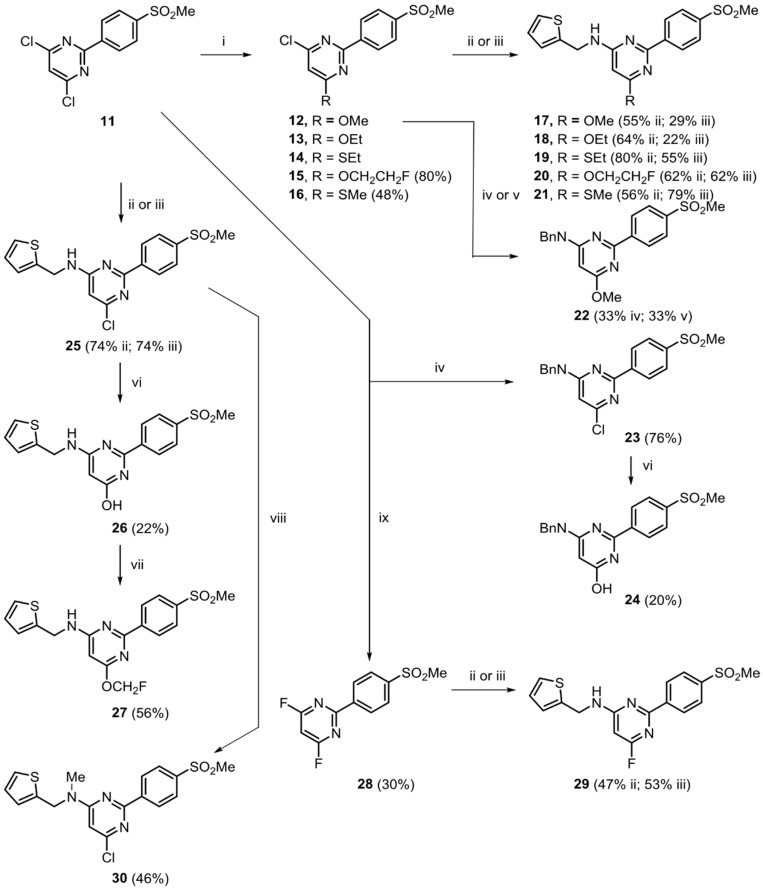
Syntheses of known and potential inhibitors, and precursors for radiolabeling. Reagents and conditions: (i) for **12**, **13**, and **15**, RX, NaH, THF, RT, 3 h; for **14** (R = SEt) and **16** (R = SMe): RNa, THF 50 °C, 3 h. (ii) thiophen-2-ylmethanamine, NEt_3_, MeCN, 5 d. (iii) thiophen-2ylmethanamine, Cs_2_CO_3_, DMF, 160 °C, 100 W, 25 min. (iv) benzylamine, NEt_3_, MeCN, 5 d. (v) benzylamine, Cs_2_CO_3_, DMF, 160 °C, 100 W, 25 min. (vi) BippyPhos (8 mol %), Pd_2_dba_3_ (2 mol %), CsOH·H_2_O, 100 °C, dioxane, 20 h. (vii) FCH_2_I, K_2_CO_3_, DMF, 40 °C, 4 h. (viii) NaH, DMF, MeI, RT, 24 h. (ix) KF, 18-crown-6, MeCN, RT, 24 h.

Scheme 1 summarizes the chemistry used to prepare known COX-2 inhibitors, potentially new inhibitors, and precursors for radiolabeling. A key synthesis material, 4,6-dichloro-2-(4(methylsulfonyl)phenyl)pyrimidine (**11**), was prepared in five steps, essentially as already described [35], starting with a Pinner reaction [36] on 4-(methylthio)benzonitrile, followed by amination, cyclization, chlorination, and oxidation. Compound **11** was readily converted into three known inhibitors [35], namely the two ethers, 4-chloro-6-methoxy-2-(4-(methylsulfonyl)phenyl)pyrimidine (**12**) and 4-chloro-6-ethoxy-2-(4-(methylsulfonyl)phenyl)pyrimidine (**13**), plus the thioether 4-chloro-6-(ethylthio)-2-(4-(methylsulfonyl)phenyl)pyrimidine (**14**), each by treatment with the respective alkoxide. The analogous 4-chloro-6-(2-fluoroethoxy) (**15**) and 4-chloro-6-thiomethyl ethers (**16**) were prepared similarly in high (80%) and moderate (48%) yield, respectively. Treatment of this set of five ethers with thiophen-2-ylmethanamine under lengthy conditions (120 h) [35], or with our newly devised and very brief (25 min) microwave method, gave the *N*-(thiophen-2-ylmethyl)pyrimidin-4-amines **17**–**21** as some of the known and prospective inhibitor targets. In two cases, the yields from the non-optimized microwave-method matched (**20**) or surpassed those from the non-microwave method (**21**). Yields of the five *N*-(thiophen-2-ylmethyl)pyrimidin-4-amines by either method ranged from moderately low (22%) to high (80%). Treatment of **12** with benzylamine instead of thiophen-2-ylmethanamine under either set of reaction conditions gave 33% yield of target inhibitor **22**. Treatment of the dichloro compound **11** with benzylamine under the non-microwave conditions gave inhibitor **23** in high yield (76%). The microwave method was not tested for this compound. Likewise, treatment of **11** with thiophen-2ylmethanamine gave ligand **25** in high yield (74%) by either method.

Treatment of **11** with potassium fluoride [37] replaced both chloro groups to give the difluoro analogue **28** in 30% yield. Treatment of **28** with thiophen-2-ylmethanamine under microwave conditions then gave the fluoro compound **29** in 53% yield, a slightly higher yield than obtained under the slower non-microwave conditions (47%).

The 4-chloro compounds **23** and **25** were readily converted with cesium hydroxide under palladium(II)-mediated conditions [38] into the respective 4-hydroxy analogues, **24** and **26**, in low but useful yields (20% and 22%, respectively). Treatment of **26** with fluoroiodomethane under basic conditions gave the 4-fluoromethyl compound **27** in 56% yield. Finally, treatment of **25** with iodomethane with sodium hydride in DMF gave the *N*-methyl derivative **30** in 46% yield.

### 2.3. COX-1 and COX-2 Inhibition Assays

Species differences in the structures of COXs are well documented, especially between rodent and human [39]. Therefore, the choice of pre-clinical species for evaluation of prospective PET radioligands must be made with care. We aimed to use rhesus monkey if inhibitors from the 2-(4-methylsulfonylphenyl)pyrimidine class showed very similar COX inhibitory potency between rhesus monkey and human. The 2-(4-methylsulfonylphenyl)pyrimidines of interest were tested for COX-1 and COX-2 inhibitory potencies using fresh monkey and human blood (Table 2). For compounds whose potencies for inhibiting human COX-1 and COX-2 had already been reported (17–19, 22, 24, 25) [35], our assay results were in fair agreement for rank order of inhibitory potency, except that we obtained somewhat higher *IC*_50_ values for human COX-2 inhibitory potency. None of the tested compounds showed monkey or human COX-1 inhibitory *IC*_50_’s below 1 nM, in accord with the observations of Orjales et al. [35] for all compounds in this class. These compounds appear to be too bulky for strong binding to the COX-1 isoform.

The thioethyl compound **19** and the hydroxy compound **26** showed much lower inhibitory potency for monkey COX-2 than that reported for human COX-2 [35]. Nevertheless, in several other cases (**17**, **20**–**22**, **24**, **27**) inhibitory potencies for the monkey COX enzymes were found to be very close to those for the human enzymes. Therefore, we concluded that monkey could indeed serve as a useful preclinical model for testing candidate PET radioligands for imaging COX-2.

Among the *N*-(thiophen-2-ylmethyl)pyrimidin-4-amines, monkey and human COX-2 inhibitory potency depended on compound structure in the following manner. Compounds containing an electron-donating group in the R^1^ position of the general structure shown in Table 2, such as OMe (**17**), OEt (**18**), SEt (**19**), OCH_2_CH_2_F (**20**), SMe (**21**), or OCH_2_F (**27**), showed high COX-2 inhibitory potencies with *IC*_50_’s between 1 and 5 nM. Compounds containing relatively small electron withdrawing groups, such as Cl (**25**) or F (**29**), in the R^1^ position also showed quite high COX-2 inhibitory potency (*IC*_50_, 3–4 nM). *N*-Methylation of **25** effectively abolished inhibitory potency at human COX-2. Replacement of the 2-thienyl group of **17** with a phenyl group as in **22** resulted in decreased COX-2 inhibitory potency. Compounds **17**, **20**, and **27** showed the highest inhibitory potencies for COX-2 in both species. The phenols, **24** and **26**, which were potentially useful as precursors for radiolabeling, showed low inhibitory potency for COX-2 [35].

### 2.4. Pharmacological Screening

At 10 μM concentration, inhibitors **17**, **18**, **27**, and **30** showed less than 50% inhibition of radioligand binding at all tested receptors, transporters, and binding sites, except **17** (50, 50.6 and 53.7% inhibition at 5-HT1_B_, benzodiazepine (BzP), and DAT (dopamine transporter), respectively), and **27** (50.8% inhibition at BzP). Therefore, this class of COX-2 inhibitor was generally devoid of very high binding affinity at any of the tested off-target sites, as is desirable for an effective PET radioligand.

### 2.5. Selection of Candidates for Radiolabeling

Inhibitors **17**, **20**, and **27** were selected from the prepared set (Table 2) for radiolabeling because they presented the highest inhibitory potencies for human and monkey COX-2, very low inhibitory potency for human and monkey COX-1, computed lipophilicities in a range generally acceptable for PET radioligands, and readily accessible positions for labeling with ^11^C (**17**) or ^18^F (**20** and **27**).

### 2.6. Radiochemistry

**Scheme 2:**
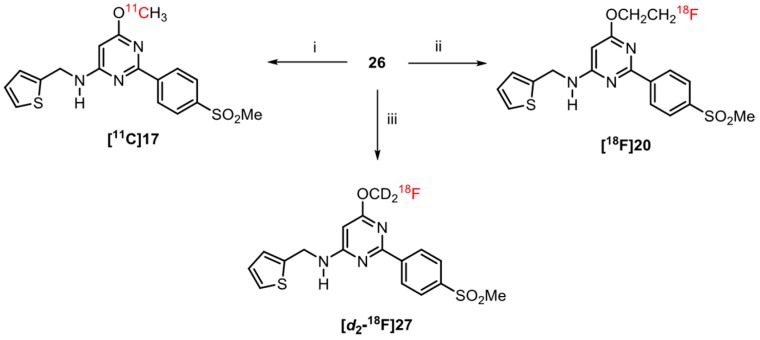
Radiosyntheses of [^11^C]17, [^18^F]20, and [^18^F]27. Reagents and conditions: (i) [^11^C]CH_3_I, DMF, TBAH, RT, 5 min. (ii) [^18^F]FCH_2_CH_2_Br, DMF, Cs_2_CO_3_, 18-crown-6, 110 °C, 15 min. (iii) [^18^F]FCD_2_Br, Cs_2_CO_3_, 18-crown-6, 110 °C, 15 min.

Three radioligands, **[^11^C]17**, **[^18^F]20**, and **[*d*_2_-^18^F]27**, were successfully prepared from the phenol precursor **26** (Scheme 2). For **[^11^C]17**, the labeling agent, [^11^C]iodomethane was rapidly prepared from cyclotron-produced NCA (no-carrier-added) [^11^C]carbon dioxide by reduction to [^11^C]methane and then high temperature iodination in a commercial apparatus, as previously described [40]. Treatment of **26** with [^11^C]iodomethane under basic conditions in DMF at RT for 5 min gave **[^11^C]17** in yields of 1.85–3.11 GBq and with molar activities of 204–492 GBq/µmol (*n* = 4) after HPLC purification and formulation for sterile intravenous injection.

For **[^18^F]20**, the labeling agent [^18^F]2-fluoro-1-bromoethane was prepared from 2-bromoethyl tosylate and NCA [^18^F]fluoride ion [41]. Treatment of **26** with [^18^F]2-fluoro-1-bromoethane under basic conditions at 110 °C for 15 min enabled formulated **[^18^F]20** to be obtained in 8.4% yield from starting [^18^F]fluoride ion with a molar activity of 60 GBq/µmol.

For **[*d*_2_-^18^F]27**, the labeling agent, [*d*_2_-^18^F]fluorobromomethane, was prepared from [*d*_2_]dibromomethane and NCA cyclotron-produced [^18^F]fluoride ion, as previously described [42]. This labeling agent was double deuterated so that any derived radioligand might resist extensive radiodefluorination in vivo [43]. Treatment of **26** with [*d*_2_-^18^F]fluorobromomethane, Cs_2_CO_3_, and 18crown-6 at 110 °C for 15 min gave **[*d*_2_-^18^F]27** in 27% yield after HPLC purification and formulation for sterile intravenous injection from starting [^18^F]fluoride ion, and with a molar activity of 66 GBq/µmol.

**Figure 1:**
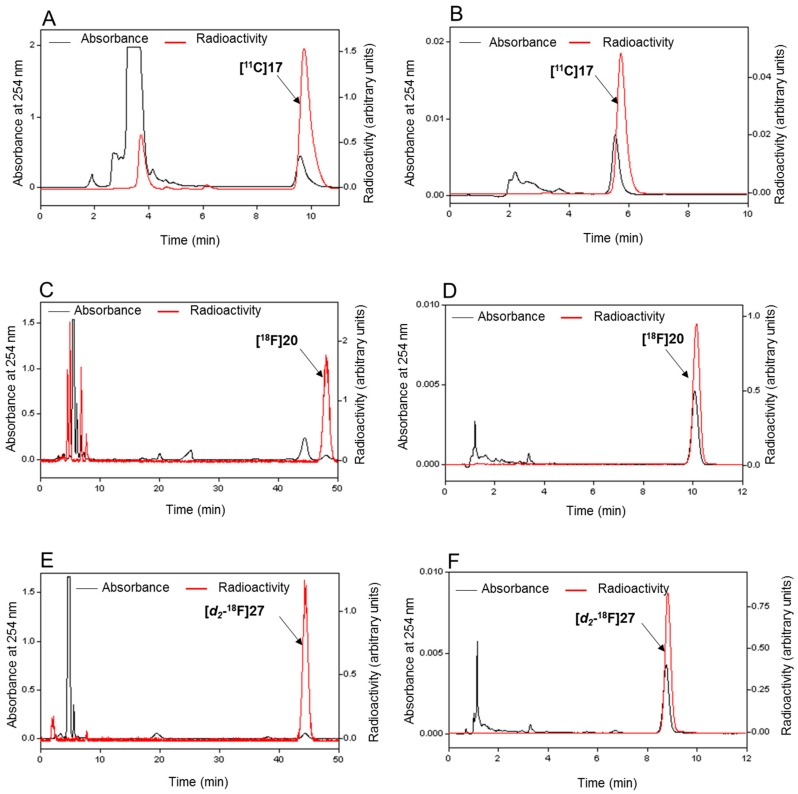
HPLC radiochromatograms for the separation of the radioligands **[^11^C]17**, **[^18^F]20**, and **[*d*_2_^18^F]27** (panels **A**, **C**, and **E**, respectively) and the respective chromatograms for HPLC analyses of the formulated radioligands (panels **B**, **D**, and **F**, respectively).

The radiochemical purities of all three radioligands exceeded 99% (Figure 1). Formulated radioligands were free of appreciable amounts of labeling precursor (**26**) or derived impurities (Figure 1). Each formulated radioligand was radiochemically stable for more than 1 h as measured by HPLC.

### 2.7. Calculated and Measured LogD for Prepared Radioligands

The measured lipophilicity values (log*D*’s) for **[^11^C]17** and **[*d*_2_-^18^F]27** were higher than the computed values by 0.94 and 0.98, respectively (Table 3). The measured value for **[^18^F]20** was almost the same as the computed value. All the measured values were within the log*D* range (2–4) observed for many effective PET radioligands for imaging proteins in brain [44], but rather higher than might be considered ideal.

## 3. Experimental

### 3.1. General

Solvents (analytical grade) and reagents were purchased from Sigma-Aldrich (St. Louis, MO, USA) unless stated otherwise. The products were isolated with flash chromatography on a CombiFlash Rf+ system (Teledyne ISCO; Lincoln NE) equipped with a silica gel cartridge (12 g, Redi*Sep^®^R*f; Isco: Lincoln, NE, USA) using hexane and EtOAc as mobile phase (general method A). If necessary to achieve purity exceeding 95%, COX-2 inhibitors were subjected to reversed phase HPLC (Beckman Coulter, Fullerton, CA, USA) on an XBridge C18 column (19 × 150 mm, 10 µm; Waters; Milford, MA, USA) eluted at 6 mL/min with a gradient mobile phase composed of aq. ammonium hydroxide (0.25 mM) (A) and acetonitrile (B) (general method B), with eluate monitored for absorbance at 254 nm. Melting points were determined on an SMP20 apparatus (Stuart; Staffordshire, UK). ^1^H (400.13 MHz), ^13^C NMR (100.62 MHz), and ^19^F NMR (376.46 MHz) spectra were recorded on an Avance 400 instrument (Bruker; Billerica, MA, USA) (see Supporting Information). ^1^H and ^13^C-NMR chemical shifts are reported in *δ* units (ppm) downfield relative to the chemical shift for tetramethylsilane. ^19^F-NMR chemical shifts were determined using the chemical shift of trifluorotoluene (−63.72 ppm) as a reference standard. Abbreviations bs, d, m, s, and t denote broad singlet, doublet, multiplet, singlet, and triplet, respectively.

LC-MS (ESI) was performed on a Velos Pro instrument (Thermo Scientific, San Jose, CA, USA) using reversed phase chromatography on a Luna C18 column (50 × 2 mm, 3 µm; Phenomenex: Torrance, CA, USA) eluted at 200 µL/min with a gradient mobile phase composed of water-methanolacetic acid (90:10:0.5 by vol.; A) and methanol-acetic acid (100:0.5, *v*/*v*; B), starting with A:B at 80:20 for 0.25 min, changed linearly to 10:90 over 3 min, and then held at 10:90 for 3.5 min (method 1). HRMS data were obtained at the Mass Spectrometry Laboratory, School of Chemical Sciences, University of Illinois at Urbana–Champaign (Urbana, IL, USA) under electron ionization conditions using a double-focusing high-resolution mass spectrometer (Micromass, Waters; Milford, MA, USA), or from the Laboratory of Bioorganic Chemistry, National Institute of Diabetes and Digestive and Kidney Diseases (Bethesda, MD, USA).

The chemical purities of all prepared inhibitors were shown to be >95% with analytical reversed phase HPLC on a Luna C18 (2) column (250 × 4.6 mm, 10 µm; Phenomenex; Torrance, CA, USA) eluted isocratically with ammonium formate (10 mM)−acetonitrile (50:50) at 2 mL/min (general method C). Eluate was monitored for absorbance at 254 nm (see Supporting Information).

cLog*D* values and p*K*a values were computed with Pallas for Windows software version 3.8 in default option (CompuDrug International; Bal Harbor, FL, USA). tPSA’s were estimated with ChemDraw version 10 (CambridgeSoft, Cambridge, MA, USA).

### 3.2. Synthesis of Target Compounds and Precursors for Radiolabeling

#### 3.2.1. Ether Derivatives of 4,6-Dichloro-2-(4-methylsulfonylphenyl)pyrimidine

*4,6-Dichloro-2-(4-methylsulfonylphenyl)pyrimidine* (**11**) was prepared in five steps from 4-(methylthio)benzonitrile and converted into the ethers 4-chloro-6-methoxy-2-(4-(methylsulfonyl)phenyl)pyrimidine (**12**) and 4-chloro-6-ethoxy-2-(4-(methylsulfonyl)phenyl)pyrimidine (**13**), and the thioether 4-chloro-6-(ethylthio)-2-(4(methylsulfonyl)phenyl)pyrimidine (**14**), all according to literature methods [35].

*4-Chloro-6-(2-fluoroethoxy)-2-(4-(methylsulfonyl)phenyl)pyrimidine* (**15**). Compound **11** (200 mg, 0.659 mmol), sodium hydride (25 mg, 1.04 mmol) and 2-fluoroethanol (41.5 µL, 0.715 mmol) in THF (1.5 mL) was added to an oven-dried pressure tube and kept at RT (room temperature) for 3 h. The solvent was evaporated off under reduced pressure. The residue was quenched with water (5 mL) and extracted with EtOAc (5 mL × 3). The organic layers were combined, washed with brine-water (1:1 *v*/*v*: 15 mL × 2), dried (MgSO_4_) and concentrated under reduced pressure. The residue was purified with flash chromatography (general method A) to give crude product (176 mg, 80%). This product was further purified with reversed phase HPLC (general method B) using a gradient mobile phase composed of aq. ammonium hydroxide (0.25 mM) (C) and acetonitrile (D), starting at 20% D and rising linearly to 95% D at 60 min to give **15** as an off-white solid; mp: 158.5–160.1 °C; ^1^H NMR (CDCl_3_): *δ* 3.11 (s, 3H), 4.82 (s, 2H), 4.84 (m, 1H), 4.88 (m, 1H), 6.83 (s, 1H), 8.04 (d, *J* = 8.65 Hz, 2H), 8.59 (d, *J* = 8.68 Hz, 2H); ^13^C NMR (CDCl_3_): *δ* 170.02, 162.56, 161.59, 142.75, 140.90, 129.37, 127.59, 127.12, 106.76, 82.01, 80.30, 66.29, 66.08, 55.40, 44.47; ^19^F NMR (CDCl_3_): *δ* −174.89; HRMS (ESI) *m*/*z* [M + H]^+^: calcd. for: C1_3_H_12_ClFN_2_O_3_S: 331.0319, found 331.0321 (Supplementary Material).

*4-Chloro-6-(methylthio)-2-(4-(methylsulfonyl)phenyl)pyrimidine)* (**16**). Sodium methanethiolate (36.4 mg, 0.519 mmol) and THF (6.0 mL) were loaded into a Schlenk tube (25-mL volume) under argon. A solution of **11** (166 mg, 0.547 mmol) in THF (1.0 mL) was then added dropwise. The Schlenk tube was sealed and heated at 50 °C for 3 h. The mixture was then quenched with water (10 mL), extracted with EtOAc (10 mL × 3) and washed with aq. NaHCO_3_ (0.1 M; 10 mL). The product was dried (MgSO_4_) and purified with flash chromatography (general method A; 15–80% ethyl acetate in hexane) to give **16** as a white solid (82.8 mg, 48%); mp: 160.2–161.9 °C; ^1^H NMR (CDCl_3_): *δ* = 2.69 (s, 3H), 3.11 (s, 3H), 7.18 (s, 1H), 8.05 (d, *J* = 8.52 Hz, 2H), 8.64 (d, *J* = 8.52 Hz, 2H); ^13^C NMR (CDCl_3_): *δ* = 173.07, 159.75, 142.66, 143.58, 141.11, 129.44, 127.60, 116.51, 44.49, 12.95; HRMS (ESI): *m*/*z* [M + H]^+^: calcd. for C_12_H_11_ClN_2_O_2_S 315.0029, found 315.0025.

#### 3.2.2. Conversion of 4-Halo-2-(4-(methylsulfonyl)phenyl)-pyrimidine Derivatives into 4-Alkylamino Derivatives

*General procedure A (non-microwave method) (GP1)*. This method was based on that in reference [35], as follows. A mixture of the appropriate 4-halo-2-(4-(methylsulfonyl)phenyl)-pyrimidine derivative (1 mmol) with Et3N (2–4 mmol) and the corresponding amine (2–4 mmol) in MeCN (7 mL) was stirred at RT for 5 d. Solvent was then removed under vacuum and the resulting residue purified with flash chromatography (general method A; 20–80% ethyl acetate in hexane). If needed, compounds were further purified to >95% purity with HPLC.

*General procedure B (fast microwave method) (GP2)*. The appropriate 4-halo-2-(4-(methylsulfonyl)phenyl)-pyrimidine (1 mmol), and corresponding amine (0.2–1 mmol), Cs_2_CO_3_ (30 mmol %), and DMF (1.00 mL) were added to a 10-mL microwave vial (Discover; CEM, NC) containing a stirrer bar. The sealed vial was irradiated (100 W) at 160 °C for 25 min. The vial contents were extracted with water (10 mL) and EtOAc (10 mL × 3), and then washed with aq. NaHCO_3_ (0.1 M; 10 mL). The combined organic layers were dried (silica gel) and the product isolated with flash chromatography (general method A; 20–80% ethyl acetate in hexane). If needed, compounds were further purified to >95% purity with reversed phase HPLC (general method B) on an XBridge C18 column using a gradient mobile phase composed of aq. ammonium hydroxide (0.25 mM) (C) and acetonitrile (D), starting at 20% D rising linearly to 95% D at 60 min.

*6-Methoxy-2-(4-(methylsulfonyl)phenyl)-N-(thiophen-2-ylmethyl)pyrimidin-4-amine* (**17**). GP2 on **12** (0.209 mmol; 62.6 mg) and thiophen-2-ylmethanamine gave **17** as an off-white solid (22 mg; 29%); mp 139.0–141.0 °C; lit. mp 120.0–124.1 °C [35]; ^1^H and ^13^C-NMR data matched those in the literature [35]; HPLC (general method C; *t*_R_ = 11.5 min; purity 99.4%). From GP1 (55%).

*6-Ethoxy-2-(4-(methylsulfonyl)phenyl)-N-(thiophen-2-ylmethyl)pyrimidin-4-amine* (**18**). GP1 on **13** and thiophen-2-ylmethanamine gave **18** as a white solid (68.3 mg; 64%); mp 169.2–170.4 °C; lit. mp 160–162.2 °C [35]; ^1^H and ^13^C-NMR data matched those in the literature [35]; HPLC (general method C; *t*_R_ = 12.8 min, purity 99.4%). From GP2 (22%).

*6-Thioethyl-2-(4-(methylsulfonyl)phenyl)-N-(thiophen-2-ylmethyl)pyrimidin-4-amine* (**19**). GP1 on **14** and thiophen-2-ylmethanamine gave **19** as a white solid (111 mg, 80%); mp: 160.0–162.1 °C; lit.mp 152.0–153.9 °C [35]; ^1^H and ^13^C-NMR data matched those in the literature [35]; HPLC (general method C; *t*_R_ = 4.5 min; purity 99.3%). From GP2 (55%).

*6-(2-Fluoroethoxy)-2-(4-(methylsulfonyl)phenyl)-N-(thiophen-2-ylmethyl)pyrimidin-4-amine* (**20**). GP2 on **15** and thiophen-2-ylmethanamine gave **20** as a white solid (56 mg; 62%); mp: 147.7–149.2 °C; ^1^H NMR (CDCl_3_): *δ* = 3.10 (s, 3H), 4.66–4.68 (m, 1H), 44.71–4.77 (m, 4H), 4.88 (m, 1H), 5.36 (b, s, 1H), 5.78 (s, 1H), 6.96–6.99 (m, 1H), 7.04 (d, *J* = 2.73 Hz,1H), 7.23 (dd, *J* = 1.04, 4.04 Hz, 1H), 8.00 (d, *J* = 8.52 Hz, 2H), 8.57 (d, *J* = 8.49 Hz, 2H); ^13^C NMR (CDCl_3_): *δ* = 170.02, 162.56, 161.59, 142.79, 140.90, 129.37, 127.59, 127.12, 106.76, 55.40, 44.47; ^19^F NMR (CDCl_3_): *δ* = −75.78. MS (ESI): *m*/*z* (%) [M + H]^+^ 408.1 (100); HRMS (ESI) *m*/*z* [M + H]^+^: calcd. for C_18_H_18_FN_3_O_3_S_2_: 408.0852, found 408.0856; HPLC (general method C; *t*_R_ = 12.3 min; purity 99.5%). From GP1 (62%).

*6-Thiomethyl-2-(4-(methylsulfonyl)phenyl)-N-(thiophen-2-ylmethyl)pyrimidin-4-amine* (**21**). GP2 on **16** and thiophen-2-ylmethanamine gave **21** as an off-white solid (49.0 mg, 79%); mp: 159.1–160.4 °C; ^1^H NMR (CDCl_3_): *δ* 2.58 (s, 3H), 3.08 (s, 3H), 4.81 (d, *J* = 5.45 Hz, 2H), 5.24 (br, s, 1H) 6.21 (s, 1H), 6.97–6.98 (m, 1H), 7.03–7.04 (m, 1H), 7.24 (dd, *J* = 1.2, 5.12 Hz, 1H), 8.00 (d, *J* = 8.6 Hz, 2H), 8.62 (d, *J* = 4.28 Hz, 2H); ^13^C NMR (CDCl_3_): *δ* 169.10, 161.23, 161.11, 143.12, 141.08, 129.12, 127.26, 126.94, 125.87, 125.31, 44.56, 40.36, 30.94, 12.71; HRMS (ESI): *m*/*z* [M + H]^+^ calcd. for C_17_H_17_N_3_O_2_S_3_: 392.0561, found 392.0559; HPLC (general method C; *t*_R_ = 15.1 min, purity 99.2%). From GP1 (56%).

*N-Benzyl-6-methoxy-2-(4-(methylsulfonyl)phenyl)pyrimidin-4-amine* (**22**). GP2 on **12** and benzylamine gave **22** as a white solid (33% yield): mp 133.4–134.6 °C; lit. mp 124.0–126.5 °C [35]; ^1^H and ^13^C-NMR data matched those in the literature [35]; HPLC (general method C; *t*_R_ = 13.6 min; purity 99.5%). From GP1 (33%).

*N-Benzyl-6-chloro-2-(4-(methylsulfonyl)phenyl)pyrimidin-4-amine* (**23**). GP1 on **11** and benzylamine gave **23** as a white solid (76% yield): mp 130.4–131.4 °C; lit. mp 144.0–145.7 °C [35]; ^1^H and ^13^C-NMR data matched those in the literature [35].

*General procedure for conversion of 4-chloropyrimidine derivatives into 4-hydroxypyrimidine derivatives (GP3).* The appropriate 4-chloropyrimidine derivative (1 mmol), Pd_2_dba_3_ (2 mol%), Bippyphos (8 mol%), CsOH·H_2_O (3 mmol), 1,4-dioxane (2 mL), and a stirrer bar were loaded into an oven-dried pressure-cap vial. The vial was sealed under argon. The contents were stirred vigorously at 100 °C for 20 h and then allowed to cool. The solution was made acidic (p*H* 3) with hydrochloric acid (2 M), diluted with water (5 mL), extracted with EtOAc (5 mL × 3), and dried (MgSO_4_). Product was then isolated with HPLC.

*6-(Benzylamino)-2-(4-(methylsulfonyl)phenyl)pyrimidin-4-ol* (**24**). GP3 gave **24** as a white solid (20.0 mg, 20%): mp: 287.9–289.2 °C; lit. mp > 265 °C [35]; ^1^H NMR matched those in the literature [35]; *m*/*z* (%) [M + H]^+^ 356.1 (100); HPLC (general method C; *t*_R_ = 2.8 min; purity 99.1%).

*6-Chloro-2-(4-(methylsulfonyl)phenyl)-N-(thiophen-2-ylmethyl)pyrimidin-4-amine* (**25**). GP2 gave **25** as a white solid (50 g; 74%); mp 170.0–171.2 °C; lit. mp 167.0–169.1 °C [35]; ^1^H and ^13^C-NMR data matched those in the literature [35]; HPLC (general method C; *t*_R_ = 12.8 min; purity 99.9%). From GP1 (74%).

*2-(4-(Methylsulfonyl)phenyl)-6-(thiophen-2-yl-methylamino)pyrimidin-4-ol* (**26**). GP3 gave **26** as a white solid (38.3 mg, 22%): mp 261.7–263.2; lit. mp 261.0–267.4 °C [35]; ^1^H and ^13^C-NMR data matched those in the literature [35]; HPLC (general method C; *t*_R_ = 2.7 min; purity 99.6%).

*6-(2-Fluoromethoxy)-2-(4-(methylsulfonyl)phenyl)-N-(thiophen-2-yl-methyl)pyrimidin-4-amine* (**27**). Compound **26** (66.6 mg, 0.184 mmol), K_2_CO_3_ (67.5 mg, 0.221 mmol), fluoroiodomethane (23.2 µL, 0.343 mmol) and DMF (1 mL) were added to an oven-dried pressure tube. The mixture was stirred at 40 °C for 4 h and then the solvent was evaporated off under reduced pressure. Water (10 mL) was added to the residue and the mixture extracted with EtOAc (10 mL × 3). The combined organic layers were washed with brine-water (1:1 *v*/*v*: 20 mL × 2), dried (MgSO_4_), and concentrated under reduced pressure. Flash chromatography (general method A) of the residue gave **27** as a white solid (41.2 g, 56%); mp: 176.6–178.2 °C; ^1^H NMR (CDCl_3_): *δ* = 3.06 (s, 3H), 4.81 (br, s, 2H), 5.42 (br, s, 1H), 5.84 (s,1H), 6.08 (s, 1H), 6.21 (s, 1H), 6.98 (t, *J* = 4.92 Hz,1H), 7.05 (d, *J* = 2.8 Hz,1H), 7.24 (d, *J* = 7.56 Hz, 1H), 8.01 (d, *J* = 8.44 Hz, 2H), 8.60 (d, *J* = 8.36 Hz, 2H); ^13^C NMR (CDCl_3_): *δ* = 168.10, 164.49, 161.63, 142.48, 141.82, 129.16, 127.32, 126.98, 125.91, 125.36, 96.56, 94.37, 44.53, 40.67; ^19^F NMR (CDCl_3_): *δ* = −155.09; HRMS (ESI) *m*/*z* [M + H]^+^ calcd. for C_17_H_16_FN_3_O_3_S_2_: 394.0684, found: 394.0686; HPLC (general method C; *t*_R_ = 11.9 min; purity 99.0%).

*4,6-Difluoro-2-(4-(methylsulfonyl)phenyl)pyrimidine* (**28**). Compound **11** (120 mg, 1.39 mmol), potassium fluoride (80.5 mg, 0.39 mmol), and 18-crown-6 (366 mg, 1.39 mmol) in MeCN (2 mL) were added to an oven-dried screw-cap tube under argon. The mixture was stirred at RT for 24 h, quenched with water (10 mL), and extracted EtOAc (10 mL × 3). The combined organic layers were washed with brine-water (1:1 *v*/*v*: 30 mL × 2) and the solvent was evaporated off under reduced pressure. The residue was purified with flash chromatography (general method A) to give **28** as an off-white solid (32 mg, 30%); mp: 164.5–166.2 °C; ^1^H NMR (CDCl_3_): *δ* = 3.11 (s, 3H), 6.57 (s, 1H), 8.08 (d, *J* = 6.22 Hz, 2H), 8.63 (d, *J* = 6.22 Hz, 2H); ^13^C NMR (CDCl_3_): *δ* = 173.69, 171.12, 163.88, 143.58, 139.45, 129.67, 127.82, 91.50 (t), 44.41; ^19^F NMR (CDCl_3_): *δ* = −54.91; HRMS (ESI): *m*/*z* [M + H]^+^ calcd. for C_11_H_8_F_2_N_2_O_2_S: 271.0353, found 271.0347.

*6-Fluoro-2-(4-(methylsulfonyl)phenyl)-N-(thiophen-2-ylmethyl)pyrimidin-4-amine* (**29**). Microwave reaction conditions in GP2 when applied to **28** with thiophen-2-ylmethanamine gave **29** as a white solid (53%); mp: 130.4–131.4 °C; ^1^H NMR (CDCl_3_): *δ* = 3.09 (s, 3H), 4.85 (s, b, 2H), 5.65 (s, b, 1H), 5.91 (s, 1H), 6.98–7.01 (m, 1H), 7.05 (s, 1H), 7.25 (d, *J* = 2.74 Hz,1H), 8.02 (d, *J* = 8.52 Hz, 2H), 8.58 (d, *J* = 8.37 Hz, 2H); ^13^C NMR (CDCl_3_): *δ* = 169.86, 165.53, 162.86, 162.68, 142.20, 141.73, 140.34, 129.27, 127.41, 127.05, 126.17, 125.57, 44.50, 40.73; ^19^F NMR (CDCl_3_): *δ* = −65.17; HRMS (ESI): *m*/*z* [M + H]^+^ calcd. for C_16_H_14_FN_3_O_2_S_2_: 364.0590, found 364.0585; HPLC (general method C; *t*_R_ = 3.9 min; purity 99.9%). From **28** (32 mg, 0.118 mmol) with GP1 (47%).

*6-Chloro-N-methyl-2-(4-(methylsulfonyl)phenyl)-N-(thiophen-2-ylmethyl)pyrimidin-4-amine* (**30**). Compound **25** (270 mg, 0.071 mmol), sodium hydride, (4.3 mg, 0.178 mmol; 95% dry) and DMF (1.0 mL) were added to a Schlenk tube (10-mL volume) under argon and left for 1 h. Iodomethane (6.88 µL, 0.111 mmol) was then added and the tube closed. After 24 h, the tube contents were evaporated to dryness and water (10 mL) was added. The mixture was then extracted with EtOAc (10 mL × 2). The combined organic layers were washed with brine-water (1:1 *v*/*v*: 20 mL × 2) and dried (MgSO_4_). The residue was purified with flash chromatography (general method A; 20–80% ethyl acetate in hexane; to give **30** as a white solid (13 mg, 46%); mp: 149.9–151.9 °C; ^1^H NMR (CDCl_3_): *δ* = 3.08 (s, 3H), 3.12 (s, 3H), 5.08 (br, s 2H), 6.45 (s, 1H), 6.95–6.97 (m, 1H), 7.03 (d, 1H, *J* = 2.00 Hz), 7.24 (d, 1H, *J* = 4.96 Hz), 8.00 (d, 2H, *J* = 8.44 Hz), 8.63 (d, 2H, *J* = 8.36 Hz); ^13^C NMR (CDCl_3_): *δ* = 162.63, 161.91, 160.67, 142.16, 142.06, 139.20, 129.33, 127.35, 126.74, 126.42, 125.66, 100.27, 47.95, 44.51 35.44. HRMS (ESI) *m*/*z* [M + H]^+^ calcd. for C_17_H_16_ClN_3_O_2_S_2_: 394.0451; found 394.0445. HPLC (general method C; *t*_R_ = 3.4 min, purity 99.9%).

### 3.3. COX-1 and COX-2 Inhibition Assays

Thromboxane B2 EIA kit (COX-1) and Prostaglandin E2 EIA Kit (COX-2) were purchased from Cayman Chemicals, USA [45]. Blood from non-treated rhesus monkey and healthy human subjects was used with these ELISA kits to establish the inhibitory potencies (*IC*_50_’s) of test compounds for these enzymes [46]. Thromboxane, the product of COX-1, and prostaglandin E2, the product of COX2, were measured using 96-well plates and a UV spectrophotometer. The concentration-inhibition curves were fitted into a 4-parameter non-linear regression model in GraphPad Prism to calculate the *IC*_50_ of each test compound.

### 3.4. Pharmacological Screening

Inhibitors **17**, **18**, **27**, and **30** were submitted to the National Institute of Mental Health Psychoactive Drug Screening Program (NIMH-PDSP) for assay of binding affinities to a wide range of human recombinant receptors (BZP, κ-opiate, M_1_, M_2_, M_3_, M_4_, M_5_, κ-opiate, 5-HT_1A_, 5-HT_1B_, 5-HT_1D_, 5-HT_1e_, 5-HT_2A_, 5-HT_2B_, 5-HT_2C_, 5-HT_3_, 5-HT_5A_, 5-HT_6_, 5-HT_7_, α_1A_, α_1B_, α_1D_, α_2A_, α_2B_, α_2C_, β_1_, β_2_, β_3_, and σ_2_), σ_1_ receptor (guinea pig), GABAA receptor (rat brain), transporters (DAT, noradrenalin, and serotonin), binding sites (PBR and the rat brain benzodiazepine site). Detailed assay protocols are available at the NIMH-PDSP web site (https://pdspdb.unc.edu/pdspWeb/).

### 3.5. Radiochemistry

#### 3.5.1. Radiochemistry Materials and Methods

Radioactivity from carbon-11 or fluorine-18 was measured with a calibrated dose calibrator (Atomlab 300, Biodex Medical Systems, Shirley, NY, USA) or an automatic γ-counter (Wizard 3”, 1480; PerkinElmer; Waltham, MA, USA). Radioactivity measurements were corrected for physical decay. Radiochemistry was executed in lead-shielded hot-cells for personal protection from radiation. Radioligands were separated with reversed phase HPLC (Beckman Coulter, Fullerton, CA, USA) on a Luna (C-18/2) column (10 × 250 mm, 10 µm; Phenomenex; Torrance, CA, USA). Radioligands were identified and measured for radiochemical purity with reversed phase HPLC (Beckman Coulter, Fullerton, CA, USA) on a Luna C18(2) column (4.6 × 250 mm, 10 µm). All HPLC column eluates were monitored for radioactivity and absorbance at 254 nm. Absorbance responses for analytical HPLC systems were calibrated for mass of injectates to enable molar activities [47] to be measured.

#### 3.5.2. Production of NCA [^11^C]Carbon Dioxide

NCA [^11^C]carbon dioxide (~85 GBq) was produced with a PETtrace cyclotron (GE Medical Systems; Milwaukee, WI, USA) according to the ^14^N(p,α)^11^C reaction [48] by irradiation of nitrogen gas (initial pressure, 160, psi; 75 mL volume) containing 1% oxygen with a proton beam (16.5 MeV, 45 µA) for 40 min.

#### 3.5.3. Production of [^11^C]Iodomethane

[^11^C]Iodomethane was produced from [^11^C]carbon dioxide via reduction to [^11^C]methane followed by vapor phase iodination [40]. Thus, at the end of proton irradiation, [^11^C]carbon dioxide was delivered to a PETtrace MeI Process Module (GE Medical Systems; Severna Park, MD, USA) through stainless tubing (OD 1/8 in, ID 1/16 in) over 3 min. The [^11^C]carbon dioxide was reduced to [^11^C]methane with hydrogen over heated nickel (360 °C) and trapped on molecular sieves (13X). Finally, the [^11^C]methane was recirculated over iodine at 720 °C to generate [^11^C]iodomethane, which was trapped on Porapak Q held in the recirculation path.

#### 3.5.4. Radiosynthesis of **[^11^C]17**

The phenol precursor **26** (1.01 mg, 2 µmol) was dissolved in DMF (80 µL) at 7 min before the end of radionuclide production. The solution was sonicated and then TBAH in methanol (1 M, 3.0 µL) was added. This solution was loaded into the loop (constructed of stainless steel tubing) of an AutoLoop apparatus (Bioscan; Washington, DC, USA). [^11^C]Iodomethane was then released into the loop and held for 5 min at RT. **[^11^C]17** was isolated with reversed phase HPLC on a Luna C18 column (10 × 250 mm, 10 µm) eluted with MeCN-water (55:45 *v*/*v*) at 6 mL/min (*t*_R_ = 9.9 min) (Figure 1A). The product solution was taken to dryness by rotary evaporation under reduced pressure and heat (80 °C). The radioactive residue was formulated in sterile physiological saline (3 mL) containing ethanol (1 mL) plus Tween 80 (12 mg; J.T. Baker, Phillipsburg, NJ, USA), and sterile-filtered (0.2 µm, Millex-MP, 25 mm; Millipore, Billerica, MA, USA) into a sterile, pyrogen-free dose vial containing saline (6 mL).

The identity of **[^11^C]17** (*t*_R_ = 5.4 min) was confirmed with reversed phase HPLC on a Luna C18 column (4.6 × 250 mm, 10 µm) eluted with MeCN-water (60:40 *v*/*v*) at 2 mL/min (Figure 1B), and with LC-MS (ESI) (method 1) of associated carrier [*t*_R_ = 4.85 min (*m*/*z*) [M + H]^+^: 376]. The radiochemical purity of **[^11^C]17** exceeded 99%. **[^11^C]17** was obtained in yields of 1.9–3.1 GBq with a molar activity of 204–492 GBq/µmol (*n* = 4).

#### 3.5.5. Production of NCA [^18^F]Fluoride Ion

NCA [^18^F]fluoride ion was produced with the ^18^O(p,n)^18^F reaction by irradiating ^18^O-enriched water (95 atom %; 1.8 mL) with a proton beam (14.1 MeV; 20–25 µA) from a PETtrace cyclotron.

#### 3.5.6. Radiosynthesis of **[^18^F]20**

For radioligand production, cyclotron-produced [^18^F]fluoride ion (~7.4 GBq) in [^18^O]water (0.4–0.65 mL) was delivered into a glass vial containing K 2.2.2 (5.0 mg, 13.3 µmol) and potassium carbonate (0.50 mg, 3.6 µmol) in MeCN-H_2_O (9:1 *v*/*v*; 0.1 mL). This solution was transferred to a modified version [42] of a TRACERlab FXFN module and diluted with MeCN (2 mL). The mixture was evaporated to dryness at 90 °C under reduced pressure with a nitrogen bleed. MeCN (2 mL) was again added and then evaporated to dryness. 2-Bromoethyltosylate (30 µL, 161 μmol) in 1,2dichlorobenzene-*t*-butanol (9:1 *v*/*v*, 1.0 mL) was added to the dry [^18^F]fluoride ion-K 2.2.2-K_2_CO_3_ complex, sealed and then heated at 95 °C for 15 min. The reaction vessel was then cooled to 35 °C. Nitrogen was used to carry the volatile [^18^F]2-fluorobromoethane through a silica gel SepPak Plus cartridge (Waters; Milford, MA, USA) and into a septum-sealed vial (1 mL; Alltech, Deerfield, IL, USA) containing phenol precursor **26** (0.5 mg, 1 µmol), Cs_2_CO_3_ (~2 mg, 6.1 µmol), and 18-crown-6 (~5 mg, 18.9 µmol) in DMF (0.5 mL). The vial was then heated at 110 °C for 15 min. **[^18^F]20** was separated with reversed phase HPLC on a Luna C18 column (10 × 250 mm, 10 µm) eluted with MeCN-H_2_O (40:60 *v*/*v*) at 6 mL/min (*t*_R_ = 49.7 min) (Figure 1C). The **[^18^F]20** was captured on a C18 Sep-Pak Plus cartridge (Waters), washed with water (5 mL), eluted with ethanol (1 mL) and saline (2 mL) containing Tween 80 (12 mg; J.T. Baker), and sterile-filtered (0.2 µm, Millex-MP, 25 mm; Millipore, Billerica, MA, USA) into a sterile, pyrogen-free dose vial containing saline (7 mL). The identity and purity of **[^18^F]20** (*t*_R_ = 10.1 min) were confirmed with reversed phase HPLC on a Luna C18 column (4.6 × 250 mm, 10 µm) eluted with MeCN-water (50:50 *v*/*v*) at 2 mL/min (Figure 1D). **[^18^F]20** was obtained in 8.4% yield from [^18^F]fluoride ion with radiochemical purity exceeding 99% and a molar activity of 60 GBq/µmol.

#### 3.5.7. Radiosynthesis of **[*d*_2_-^18^F]27**

For radioligand production, cyclotron-produced [^18^F]fluoride ion (~7.4 GBq) in [^18^O]water (0.4–0.65 mL) was delivered into a glass vial containing K 2.2.2 (5.0 mg, 13.3 µmol) and potassium carbonate (0.50 mg, 3.6 µmol) in MeCN-H_2_O (0.1 mL, 9:1 *v*/*v*). This solution was transferred to a modified version [42] of a TRACERlab FXFN module (GE) and diluted with MeCN (2 mL). The mixture was evaporated to dryness at 90 °C under reduced pressure with a nitrogen flow. MeCN (2 mL) was again added and evaporated to dryness. The vessel was sealed. Then CD_2_Br_2_ (100 µL) in MeCN (1.0 mL) was added to the dry [^18^F]fluoride ion-K 2.2.2-K_2_CO_3_ complex, which was then heated at 95 °C for 15 min. The vessel was then cooled to 35 °C. Nitrogen was used to transfer the volatile [*d*_2_-^18^F]fluorobromomethane through a series of four silica gel SepPak Plus cartridges (Waters; Milford, MA) into a septum-sealed 1-mL vial (Alltech, Deerfield, IL, USA) containing phenol precursor **26** (0.5 mg, 1 µmol), Cs_2_CO_3_ (~2 mg, 6.1 µmol) and 18-crown-6 (~5 mg, 18.9 µmol) in DMF (0.5 mL). The vial was sealed and heated at 110 °C for 15 min. The **[*d*_2_-^18^F]27** was separated with reversed phase HPLC on a Luna C18 column (10 × 250 mm, 10 µm) eluted with MeCN-water (40:60 *v*/*v*) at 6 mL/min (*t*_R_ = 45 min) (Figure 1E). The **[*d*_2_-^18^F]27** was captured on a C18 Sep-Pak Plus cartridge (Waters), washed with water (5 mL), eluted with ethanol (1 mL) and saline (2 mL) containing Tween 80 (12 mg), and sterile-filtered (0.2 µm, Millex-MP, 25 mm) into a sterile, pyrogen-free dose vial containing saline (7 mL). The identity of the **[*d*_2_-^18^F]27** was confirmed with reversed phase HPLC on a Luna C18 column (4.6 × 250 mm) eluted with MeCN-water (50:50 v/v) at 2 mL/min (*t*_R_ = 9 min) (Figure 1F). **[*d*_2_-^18^F]27** was obtained in 27%yield from [^18^F]fluoride ion with radiochemical purity exceeding 99% and a molar activity of 66 GBq/µmol).

### 3.6. LogD Determinations

*c*Log*D* (at pH 7.4) values were computed with Pallas for Windows software version 3.8 in default mode (CompuDrug International; Bal Harbor, FL, USA). The log*D* values of **[^11^C]17**, **[^18^F]20**, and **[*d*_2_-^18^F]27** were measured by a method that we have described previously [49]. The radioactivity contents in the *n-*octanol and sodium phosphate buffer (0.1 M; pH 7.4) phases were determined with a γ-counter.

## 4. Conclusions

Three COX-2 inhibitors, **17**, **20**, and **27**, were identified from the 2-(4-methylsulfonylphenyl)-pyrimidine class as having promising physiochemical and pharmacological properties for development as PET radioligands for imaging COX-2 in monkey and human in vivo. Methods were established for satisfactorily labeling these ligands with positron-emitters for comparative PET imaging in monkey in vivo, including a neuroinflammation model [50]. These results will be published in detail elsewhere.

## Figures and Tables

**Table 1:** Physicochemical, structural and previously reported pharmacological parameters for selected COX-2 inhibitors.

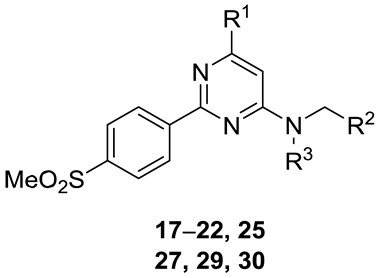										
#	R^1^	R^2^	R^3^	clog*D*	tPSA (Å^2^)	HBD	Hetero-Atoms	M.Wt. (Da)	HWB ^a^ *IC*_50_ (nM)	Selectivity vs. COX-1 ^b^
**17**	OMe	2-Thi ^c^	H	2.80	80.1	1	8	375	0.4	>22,004
**18**	OEt	2-Thi	H	2.88	80.1	1	8	389	0.3	>33,333
**19**	SEt	2-Thi	H	3.47	70.9	1	8	405	1.2	>8403
**20**	O(CH_2_)_2_F	2-Thi	H	3.09	80.1	1	9	407		
**21**	SMe	2-Thi	H	3.34	70.9	1	8	391		
**22**	OMe	Ph	H	2.71	80.1	1	7	369	5.1	>19,646
**25**	Cl	2-Thi	H	2.74	70.9	1	8	379	1.2	>81,300
**27**	OCH_2_F	2-Thi	H	2.72	80.2	1	9	393		
**29**	F	2-Thi	H	2.91	70.9	1	8	363		
**30**	Cl	2-Thi	Me	3.47	62.1	0	8	394		
**Optimal parameter space**				2–3	<90	low	<9	<500	low nM	>100

^a^ HWB = Human whole blood. ^b^ Data from reference [35]. ^c^ 2-Thi = 2-thienyl. SEM’s of values are <15%.

**Table 2:** Structures and *IC*_50_’_s_ of inhibitors **17**–**27**, **29**, and **30** for COX-1 and COX-2 in monkey and human whole blood (WB).

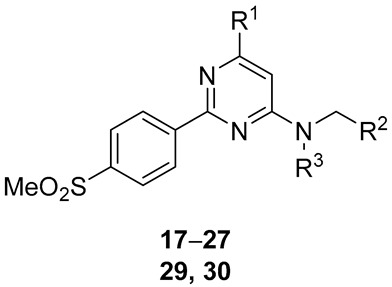							
#	R^1^	R^2^	R^3^	*IC*_50_ (nM) ^a^			
				COX-1		COX-2	
				Monkey WB	Human WB	Monkey WB	Human WB
**17**	OMe	2-Thi ^b^	H	>1000	>1000 >8800 ^c^	2.58 ± 0.23	2.89 ± 0.15 0.4 ^c^
**18**	OEt	2-Thi	H		>10,000 ^d^ >10^5 c^		3 ^d^ 0.3 ^c^
**19**	SEt	2-Thi	H	>1000	>1000 >10,000 ^c^	52.3 ± 0.01	3.52 ± 0.43 1.2 ^c^
**20**	O(CH_2_)_2_F	2-Thi	H			1.28 ^e^	1.9 ^e^
**21**	SMe	2-Thi	H	>1000	>1000	4.83 ± 2.31	2.65 ± 0.45
**22**	OMe	Ph	H	>1000	>1000 >10^5 c^	14.6 ± 10.1	7.35 ± 1.35 5.1 ^c^
**23**	Cl	Ph	H		>10^5 c^		10.9*^c^*
**24**	OH	Ph	H	>1000	>1000 >10^5 c^	> 1000 ^d^	>1000 ^d^ 49.3 ^c^
**25**	Cl	2-Thi	H		>10,000 ^d^ >10^5 c^		4 ^d^ 1.2 ^c^
**26**	OH	2-Thi	H		>10^5 c^	10 ^d^	79.4 ^c^
**27**	OCH_2_F	2-Thi	H	>1000	>1000	2.26 ± 0.45	2.26 ± 0.63
**29**	F	2-Thi	H				2.5 ^d^
**30**	Cl	2-Thi	Me		> 10^8 d^		>1000

^a^ Data are for mean ± SD from triplicate measurements, unless otherwise stated. ^b^ 2-Thi = 2-thienyl ^c^ Data from reference [35]. SE of values were <15%. ^d^ n = 1. ^e^ n = 2.

**Table 3:** Computed and measured log*D* values for radioligands.

Radioligand	*c*Log*D*	Log*D* (*n* = 6)
**[^11^C]17**	2.80	3.74 ± 0.02
**[^18^F]20**	3.09	2.94 ± 0.32
**[*d*_2_-^18^F]27**	2.72	3.70 ± 0.12

## Supplementary Materials

NMR spectra and HPLC analyses of synthesis intermediates and new COX-2 inhibitors.

## References

[1] Seibert K., Zhang Y., Leahy K., Hauser S., Masferrer J., Perkins W., Lee L., Isakson P. (1994). Pharmacological and biochemical demonstration of the role of cyclooxygenase 2 in inflammation and pain. Proc. Natl. Acad. Sci. USA.

[2] Phillis J.W., Horrocks L.A., Farooqui A.A. (2006). Cyclooxygenases, lipoxygenase, and epoxygenases in CNS: Their role and involvement in neurological disorders. Brain Res. Rev..

[3] Hong H., Kim B.S., Im H.-I. (2016). Pathophysiological role of neuroinflammation in neurodegenerative diseases and psychiatric disorders. Int. Neurourol. J..

[4] Choi S.-H., Aid S., Bosetti F. (2009). The distinct roles of cyclooxygenase-1 and -2 in neuroinflammation: Implications for translational research. Trends Pharmacol. Sci..

[5] Eberstål S., Sandén E., Fritzell S., Darabi A., Visse E., Siesjö P. (2014). Intratumor COX-2 inhibition enhances GM-CSF immunotherapy against established mouse GL261 brain tumor. Int. J. Cancer.

[6] Zweers M.C., de Boer T.N., van Roon J., Bijlsma J.W.J., Lafeber F.P.J.G., Mastbergen S.C. (2011). Celecoxib: Considerations regarding its potential disease-modifying properties in osteoarthritis. Arthritis Res. Ther..

[7] Yermakova A., O’Banion M.K. (2000). Cyclooxygenases in the central nervous system: Implications for treatment of neurological disorders. Curr. Pharm. Des..

[8] Hoozemans J.J.M., Veerhuis R., Janssen I., van Elk E.-J., Rozemuller A.J.M., Eikelenboom P. (2002). The role of cyclooxygenase-1 and -2 activity in prostaglandin E2 secretion by culture human adult microglia: Implications for Alzheimer’s disease. Brain Res..

[9] Teismann P., Tieu K., Choi D.-K., Wu D.-C., Naini A., Hunot S., Vila M., Jackson-Lewis V., Przedborski S. (2003). Cyclooxygenase-2 is instrumental in Parkinson’s disease neurodegeneration. Proc. Natl. Acad. Sci. USA.

[10] Garavito M.R. (1996). The cyclooxygenase-2 structure: New drugs for an old target?. Nat. Struct. Biol..

[11] Luong C., Miller A., Barnett J., Chow J., Ramesha C., Browner M.F. (1996). Flexibility of the NSAID binding site in the structure of human cyclooxygenase-2. Nat. Struct. Biol..

[12] Bakhle Y.S. (1999). Structure of COX-1 and COX-2 enzyme and their interaction with inhibitors. Drugs Today.

[13] Plount Price M.L., Jorgensen W. (2000). Analysis of binding inhibitory potencies of celecoxib analogues with COX-1 and COX-2 from combined docking and Monte Carlo simulation and insight into the COX-2/COX-1 selectivity. J. Am. Chem. Soc..

[14] Habeb A.G., Rao P.N.R., Knaus E.E.W. (2000). Design and syntheses of diarylisoxazoles: Novel inhibitors of cyclooxygenase-2 (COX-2) with analgesic antiinflammatory activity. Drug Dev. Res..

[15] Penning T., Talley J.J., Bertenshaw S., Carter J., Collins P., Docter S., Graneto M., Lee L., Malecha J., Miyashiro J. (1997). Synthesis and biological evaluation of the 1,5-diarylpyrazole class of cyclooxygenase-2 inhibitors: Identification of 4-[5-(4-methylphenyl)-3-(trifluoromethyl)-1*H*-pyrazol-1yl]benzenesulfonamide (SC-58635, Celecoxib). J. Med. Chem..

[16] Desmond R., Dolling U., Marcune B., Tillyer R., Tschaen D. (1996). Process for making phenylheterocycles useful as COX-2 inhibitors. World Patent.

[17] Talley J.J., Brown D.L., Carter J.S., Graneto M.J., Koboldt C.M., Masferrer J.L., Perkins W.E., Rogers R.S., Shaffer A.F., Zhang Y.Y. (2000). 4-[5-Methyl-3-phenylisoxazol-4-yl]-benzenesulfonamide, Valdecoxib: A potent and selective inhibitor of COX-2. J. Med. Chem..

[18] Prabhakara J., Majo V.J., Simpson N.R., Van Heertum R.L., Mann J.J., Kumar J.S.D. (2005). Synthesis of [^11^C]celecoxib: A potential PET probe for imaging COX-2 expression. J. Label. Comp. Radiopharm..

[19] Ji B., Kumata K., Onoe H., Kaneko H., Zhang M.R., Seki C., Ono M., Shukuri M., Tokunaga M., Minamihisamatsu T. (2013). Assessment of radioligands for PET imaging of cyclooxygenase-2 in an ischemic neuronal injury model. Brain Res..

[20] Prabhakaran J., Underwood M.D., Parsey R.V., Arango V., Majo V.J., Simpson N.R., Van Heertum R., Mann J.J., Kumar J.S.D. (2007). Synthesis and in vivo evaluation of [^18^F]-4-[5-(4-methylphenyl)-3(trifluoromethyl)-1*H*-pyrazol-1-yl]benzenesulfonamide as a PET imaging probe for COX-2 expression. Bioorg. Med. Chem. Lett..

[21] Fujisaki Y., Kawamura K., Wang W.-F., Ishiwata K., Yamamota F., Kuwano T., Ono M., Maeda M. (2005). Radiosynthesis and in vivo evaluation of ^11^C-labeled 1,5-diarylpyrazole derivatives for mapping cyclooxygenases. Ann. Nucl. Med..

[22] Kaur J., Tietz O., Bhardwaj A., Marshall A., Way J., Wuest M., Wuest F. (2015). Design, synthesis, and evaluation of an ^18^F-labeled radiotracer based on celecoxib–NBD for positron emission tomography (PET) imaging of cyclooxygenase-2 (COX-2). ChemMedChem.

[23] De Vries E.F.J., Doorduin J., Dierckx R.A., van Waarde A. (2008). Evaluation of [^11^C]rofecoxib as PET tracer for cyclooxygenase 2 overexpression in rat models of inflammation. Nucl. Med. Biol..

[24] Comley R.A., Passchier J., Willemsen A., Wall A., Bergstrom M., Langstrom B., Pruim J., Wishart M., Rabiner E., Gunn R.N. (2010). Uptake and regional distribution of [^11^C]rofecoxib in human brain. NeuroImage.

[25] Toyokuni T., Kumar J.S.D., Walsh J.C., Shapiro A., Talley J.J., Phelps M.E., Herschman H.R., Barrio J.R., Satymurthy N. (2005). Synthesis of 4-(5-[^18^F]fluoromethyl-3-phenylisoxazol-4-yl)benzenesulfonamide, a new [^18^F]fluorinated analogue of valdecoxib, as a potential radiotracer for imaging cyclooxygenase-2 with positron emission tomography. Bioorg. Med. Chem. Lett..

[26] Tanaka M., Fujisaka Y., Kawamura K., Ishiwata K., Qinggeletu F.Y., Mukai T., Maeda M. (2006). Radiosynthesis and evaluation of ^11^C-labeled diaryl-substituted imidazole and indole derivatives for mapping cyclooxygenase-2. Biol. Pharm. Bull..

[27] Wuest F., Kniess T., Bergmann R., Pietszsch J. (2008). Synthesis and evaluation in vitro and in vivo of a ^11^C-labeled cyclooxygenase-2 (COX-2) inhibitor. Bioorg. Med. Chem..

[28] De Vries E.F.J., van Waarde A., Buursma A.R., Vaalburg W. (2003). Synthesis and in vivo evaluation of ^18^F-desbromo-DuP-697 as a PET tracer for cyclooxygenase-2 expression. J. Nucl. Med..

[29] Leblanc Y., Guathier J., Ethier D., Guay J., Mancinin J., Reindeau Tagari P., Vickers P., Wong E., Prasit P. (1995). Synthesis and biological evaluation of 2,3-diarylthiophenes as selective COX-2 and COX-1 inhibitors. Bioorg. Med. Chem. Lett..

[30] Tietz O., Wuest M., Marshall A., Gubrecht D., Hamman I., Wang M., Bergman C., Way J.D., Wuest F. (2016). PET imaging of cyclooxygenase-2 (COX-2) in a preclinical colorectal cancer model. EJNMMI Res..

[31] Laube M., Kniess T., Pietzsch J. (2013). Radiolabeled COX-2 inhibitors for non-invasive visualization of COX-2 expression and activity—A critical update. Molecules.

[32] Pike V.W. (2016). Considerations in the development of reversibly binding PET radioligands for brain imaging. Curr. Med. Chem..

[33] Pike V.W. (2009). PET Radiotracers: Crossing the blood–brain barrier and surviving metabolism. Trends Pharmacol. Sci..

[34] De Vries E.F. (2006). Imaging of cyclooxygenase (COX-2) expression; potential uses in diagnosis and drug evaluation. Curr. Pharm. Des..

[35] Orjales A., Mosquera R., Lopez B., Olivera R., Labeaga L., Nuῆez M.T. (2008). Novel 2-(4methylsulfonylphenyl)pyrimidine derivates as highly potent and specific COX-2 inhibitors. Bioorg. Med. Chem..

[36] Cheng C.C. (1990). Preparation of an *o*-substituted benzamidine by the Pinner method. A literature clarification. Org. Prep. Proced. Int..

[37] Pews G.R., Puckett W.E. (1989). 1,1,1-Trichloro-3-[5-(2,4,6-trifluoropyrimidyl)]-3,4-epoxybutane. J. Fluorine Chem..

[38] Cheung C.W., Buchwald S.L. (2014). Palladium-catalyzed hydroxylation of aryl and heteroaryl halides enabled by the use of a palladacycle precatalyst. J. Org. Chem..

[39] Huff R.G., Bayram E., Tan H., Knutson S.T., Knaggs M.H., Richon A.B., Santiago P., Fetrow J.S. (2005). Chemical and structural diversity in cyclooxygenase protein active sites. Chem. Diversity.

[40] Larsen P., Ulin J., Dahlström K., Jensen M. (1997). Synthesis of [^11^C]iodomethane by iodination of [^11^C]methane. Appl. Radiat. Isot..

[41] Savolainen H., Cantore M., Colabufo N.A., Elsinga P.H., Windhorst A.D., Luurtsema G. (2015). Synthesis and preclinical evaluation of three novel fluorine-18 labeled radiopharmaceuticals for P-glycoprotein PET imaging at the blood–brain barrier. Mol. Pharmacol..

[42] Chin F.T., Morse C.L., Shetty H.U., Pike V.W. (2006). Automated radiosynthesis of [^18^F]SPA-RQ for imaging human brain NK1 receptors with PET. J. Label. Comp. Radiopharm..

[43] Kuchar M., Mamat C. (2015). Methods to increase the metabolic stability of ^18^F-radiotracers. Molecules.

[44] Zoghbi S.S., Anderson K.B., Jenko K.J., Luckenbaugh D.A., Innis R.B., Pike V.W. (2012). On quantitative relationships between drug-like compound lipophilicity and plasma free fraction in monkey and human. J. Pharm. Sci..

[45] Assay Kits. https://www.caymanchem.com/app/template/Products.vm/active/kits.

[46] Singh P., Shrestra S., Cortes-Salva M.-Y., Jenko K.J., Zoghbi S.S., Morse C.L., Innis R.B., Pike V.W. (2018). 3-Substituted 1,5-diaryl-1*H*-1,2,4-triazoles as prospective PET radioligands for imaging brain COX-1 in monkey. Part 1: Synthesis and pharmacology. ACS Chem. Neurosci..

[47] Coenen H.H., Gee A.D., Adam M., Antoni G., Cutler C.S., Fujibayashi Y., Jeong J.-M., Mach R.H., Mindt T.L., Pike V.W. (2017). Consensus nomenclature rules for radiopharmaceutical chemistry—Setting the record straight. Nucl. Med. Biol..

[48] Christman D.R., Finn R.D., Karlstrøm K., Wolf A.P. (1975). The production of ultra high specific activity ^11^Clabeled hydrogen cyanide, carbon dioxide, carbon monoxide and methane via the ^14^N(p,α)^11^C reaction. Int. J. Appl. Radiat. Isot..

[49] Briard E., Zoghbi S.S., Imaizumi M., Gourley J.P., Shetty H.U., Hong J., Cropley V., Fujita M., Innis R.B., Pike V.W. (2008). Synthesis and evaluation in monkey of two sensitive ^11^C-labeled aryloxyanilide ligands for imaging brain peripheral benzodiazepine receptors in vivo. J. Med. Chem..

[50] Gallagher E., Shrestha S., Eldridge M., Cortes M., Yu Z.-X., Lehmann M., Kim M.-J., Singh P., Fredericks M., Tye G. (2018). Novel PET radioligands show that COX-2, but not COX-1, is induced by neuroinflammation in rhesus macaque. Biol. Psychiatry.

