# Supplementary material for: 2-(4-Methylsulfonylphenyl)pyrimidines as Prospective Radioligands for Imaging Cyclooxygenase-2 with PET—Synthesis, Triage, and Radiolabeling
Source: Molecules. 2018 Nov 2;23(11):2850. doi: 10.3390/molecules23112850 (PMC6278313; doi:10.3390/molecules23112850)
Supplement: Supplementary file 1 [file molecules-23-02850-s001.pdf]

molecules

Supporting Information

2-(4-Methylsulfonylphenyl)pyrimidines as prospective radioligands for imaging cyclooxygenase-2 with PET – synthesis, triage, and radiolabeling

Michelle Y. Cortes-Salva, Stal Shrestha, Prachi Singh, Cheryl L. Morse, Kimberly J. Jenko, Jose A. Montero Santamaria, Sami S. Zoghbi, Robert B. Innis, and Victor W. Pike

Molecular Imaging Branch, National Institute of Mental Health, National Institutes of Health, 10 Center Drive, Bethesda, Maryland 20892, United States

Contents

	Page
NMR spectra for hydroxy precursor **15**	S3
NMR spectra for hydroxy precursor **16**	S5
NMR spectra and HPLC analyses for COX inhibitor **17**	S6
NMR spectra and HPLC analyses new COX-2 inhibitor **20**	S8
NMR spectra and HPLC analyses new COX-2 inhibitor **21**	S10
NMR spectra and HPLC analyses new COX-2 inhibitor **26**	S12
NMR spectra and HPLC analyses new COX-2 inhibitor **27**	S14
NMR spectra and HPLC analyses new COX-2 inhibitor **28**	S16
NMR spectra and HPLC analyses new COX-2 inhibitor **29**	S18
NMR spectra and HPLC analyses new COX-2 inhibitor **30**	S20

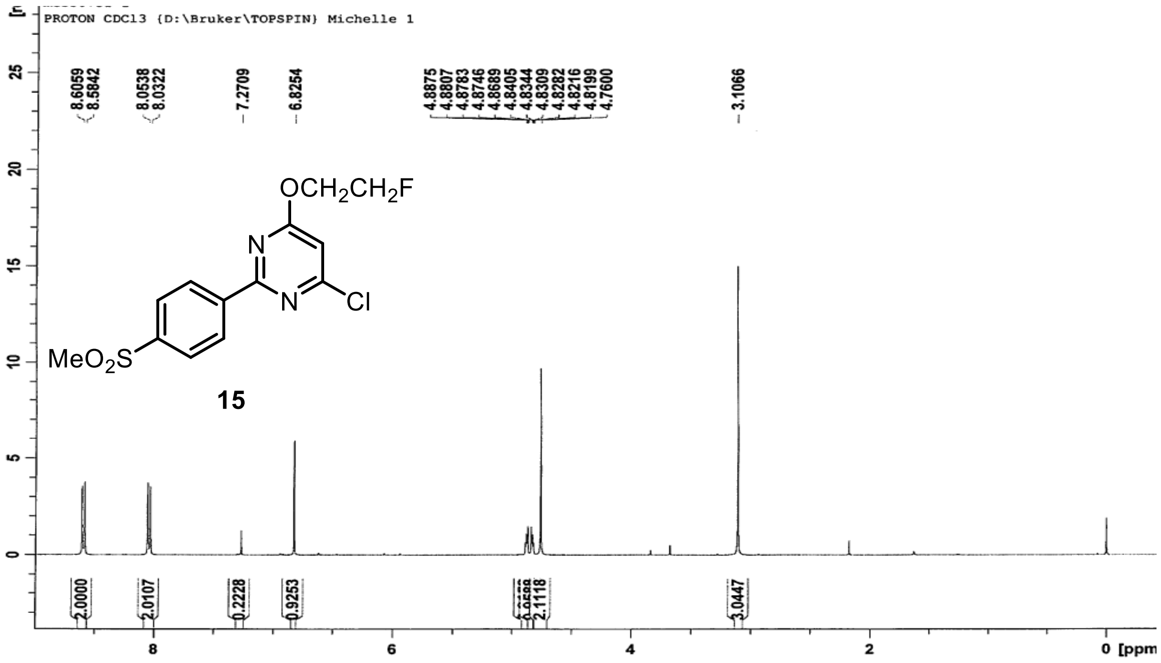
PROTON CDCl3 {D:\Bruker\TOPSPIN} Michelle 1
8.6059
8.5842
8.0538
8.0322
7.2709
6.8254
4.8875
4.8807
4.8783
4.8746
4.8689
4.8405
4.8344
4.8309
4.8282
4.8216
4.8199
4.7600
3.1066
OCH2CH2F
N
N
Cl
MeO2S
15
2.0000
2.0107
0.2228
0.9253
2.1118
3.0447
8
6
4
2
0 [ppm

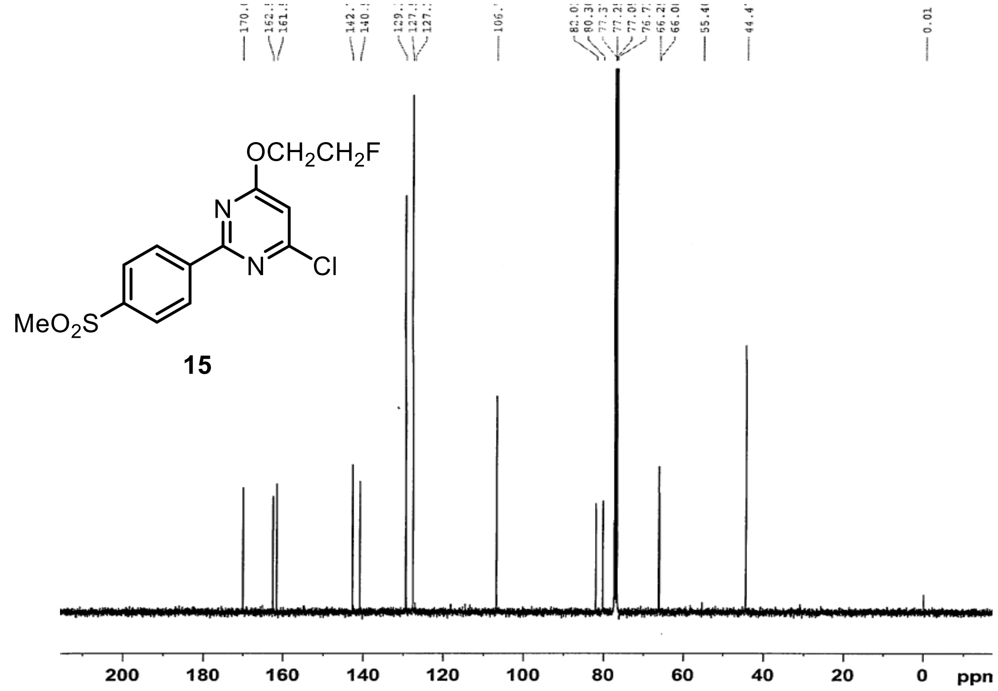
0.01
OCH2CH2F
N
N
Cl
MeO2S
15
200
180
160
140
120
100
80
60
40
20
0
ppn

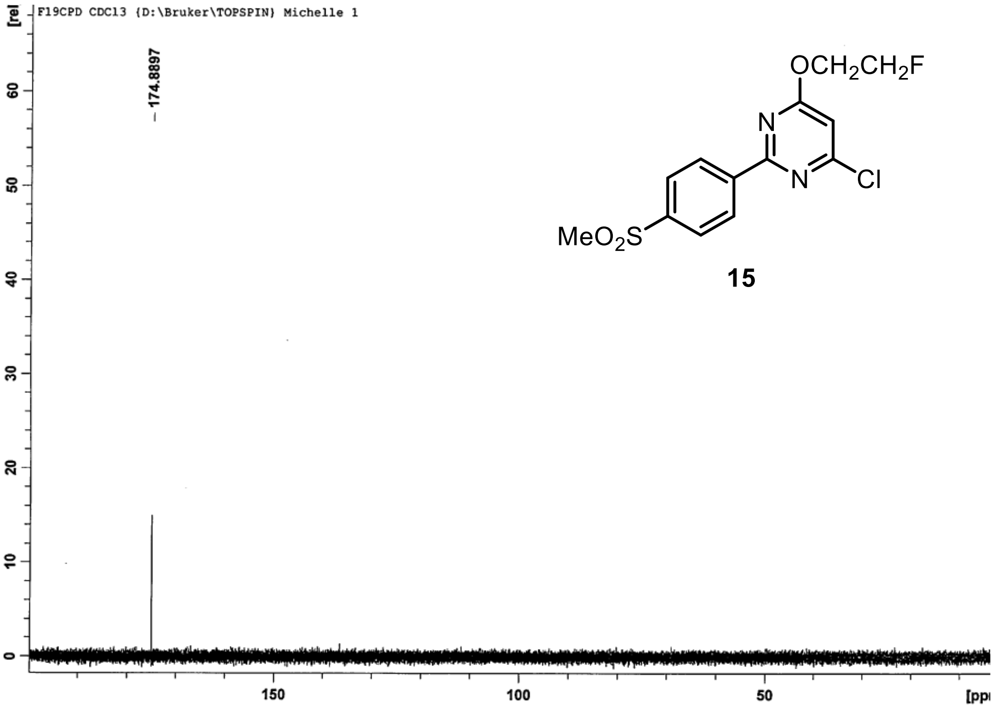
F19CPD CDCl3 {D:\Bruker\TOPSPIN} Michelle 1
-174.8897
[rel
60
50
40
30
20
10
0
150
100
50
[ppm
OCH2CH2F
N
N
Cl
MeO2S
15

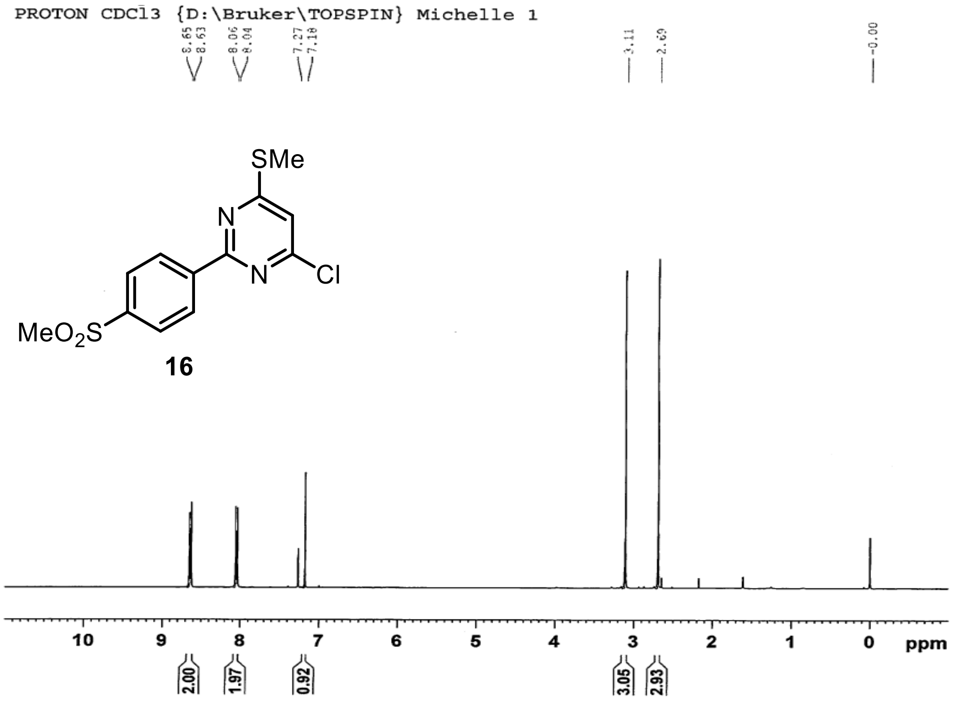
PROTON CDCl3 {D:\Bruker\TOPSPIN} Michelle 1
SMe
N
N
Cl
MeO2S
16
10
9
8
7
6
5
4
3
2
1
0
ppm
2.00
1.97
0.92
3.05
2.93

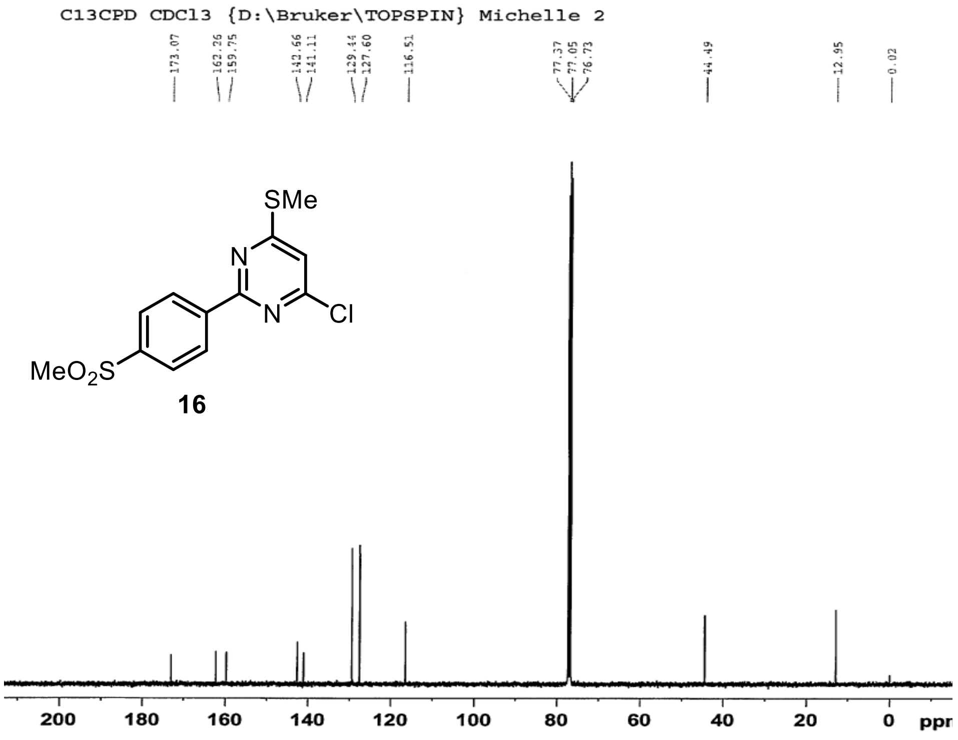
C13CPD CDCl3 {D:\Bruker\TOPSPIN} Michelle 2
173.07
162.26
159.75
142.66
141.11
129.44
127.60
116.51
77.37
77.05
76.73
44.49
12.95
0.02
SMe
N
N
Cl
MeO2S
16
200
180
160
140
120
100
80
60
40
20
0

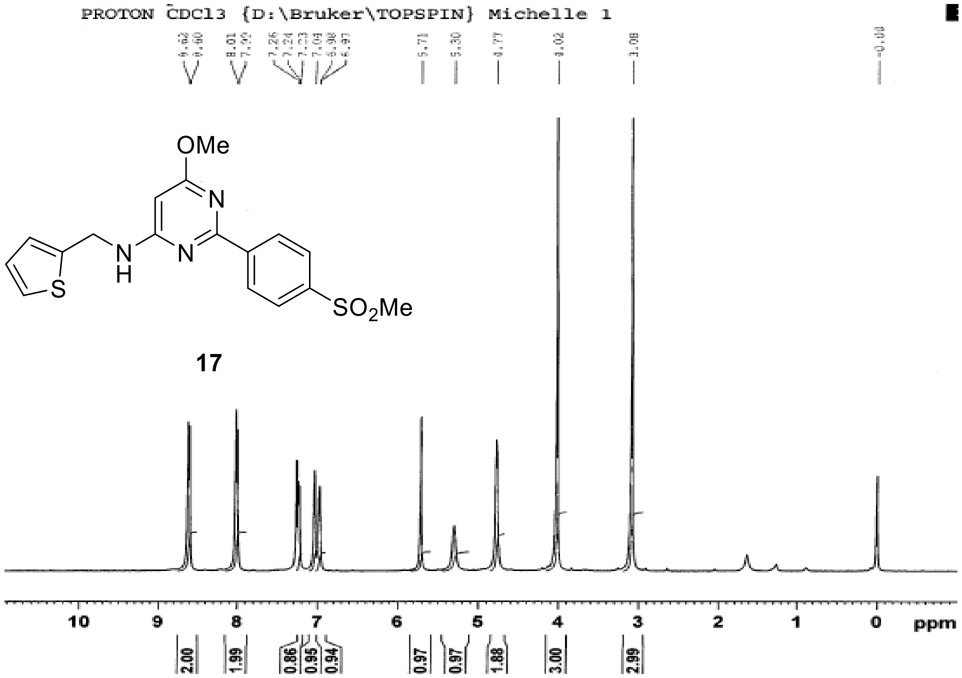
PROTON CDCl3 {D:\Bruker\TOPSPIN} Michelle 1
OMe
N
N
NH
S
SO2Me
17
ppm
2.00
1.99
0.86
0.95
0.94
0.97
0.97
1.88
3.00
2.99

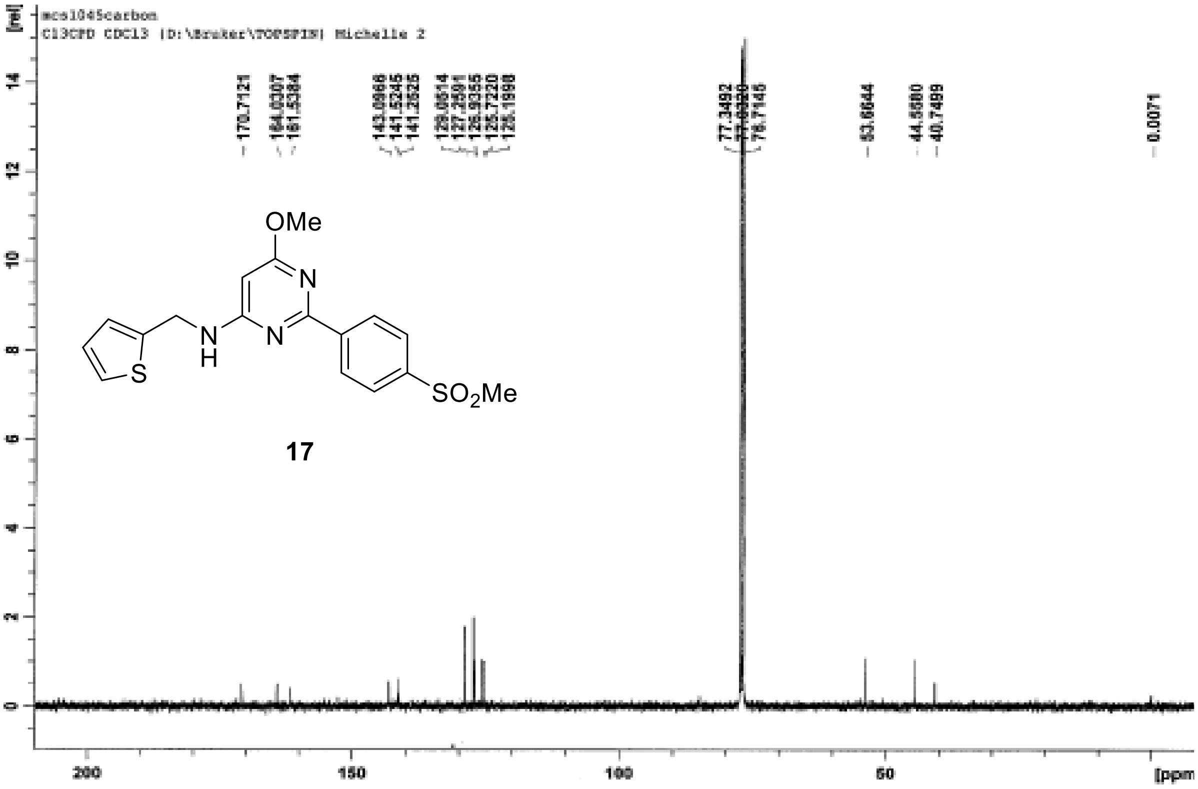
mcsl045carbon
C13CPD CDCl3 {D:\Bruker\TOPSPIN} Michelle 2
170.7121
164.0307
161.5384
143.0966
141.5245
141.2625
129.0514
127.2591
126.9355
125.7220
125.1990
77.3492
76.7145
53.6644
44.5580
40.7499
-0.0071
OMe
N
N
NH
S
SO2Me
17
[rel]
[ppm

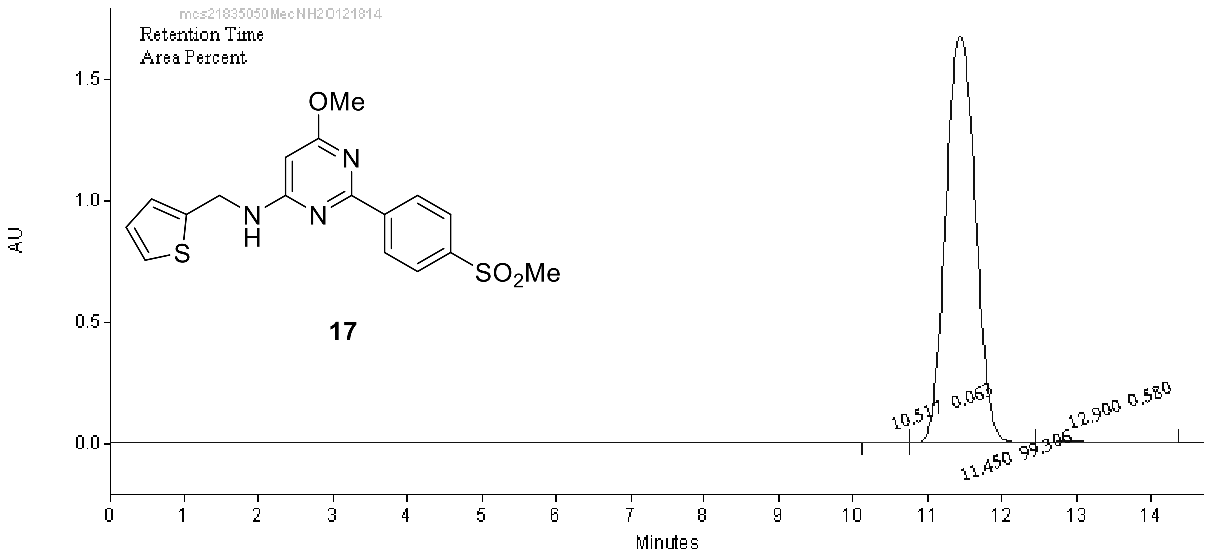

mcs21835050MecNH2O121814
Retention Time
Area Percent
OMe
N
N
N
H
S
SO2Me
17
10.517 0.063
11.450 99.306
12.900 0.580
AU
Minutes

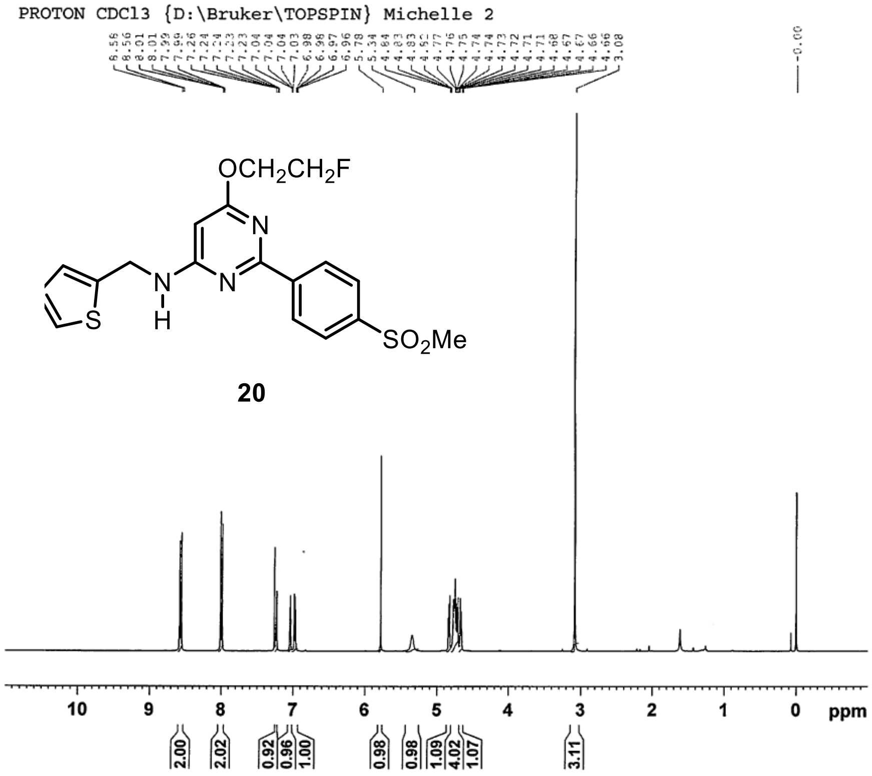
PROTON CDCl3 {D:\Bruker\TOPSPIN} Michelle 2
OCH2CH2F
SO2Me
20
ppm

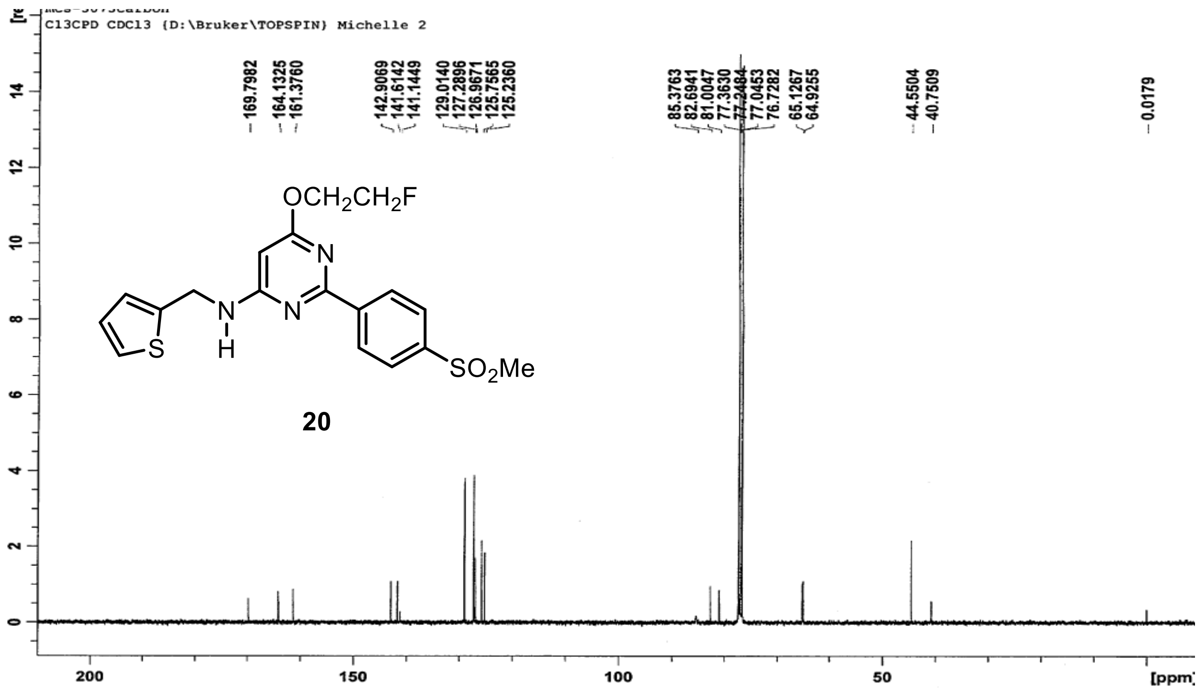
C13CPD CDCl3 {D:\Bruker\TOPSPIN} Michelle 2
OCH2CH2F
SO2Me
20
[ppm]

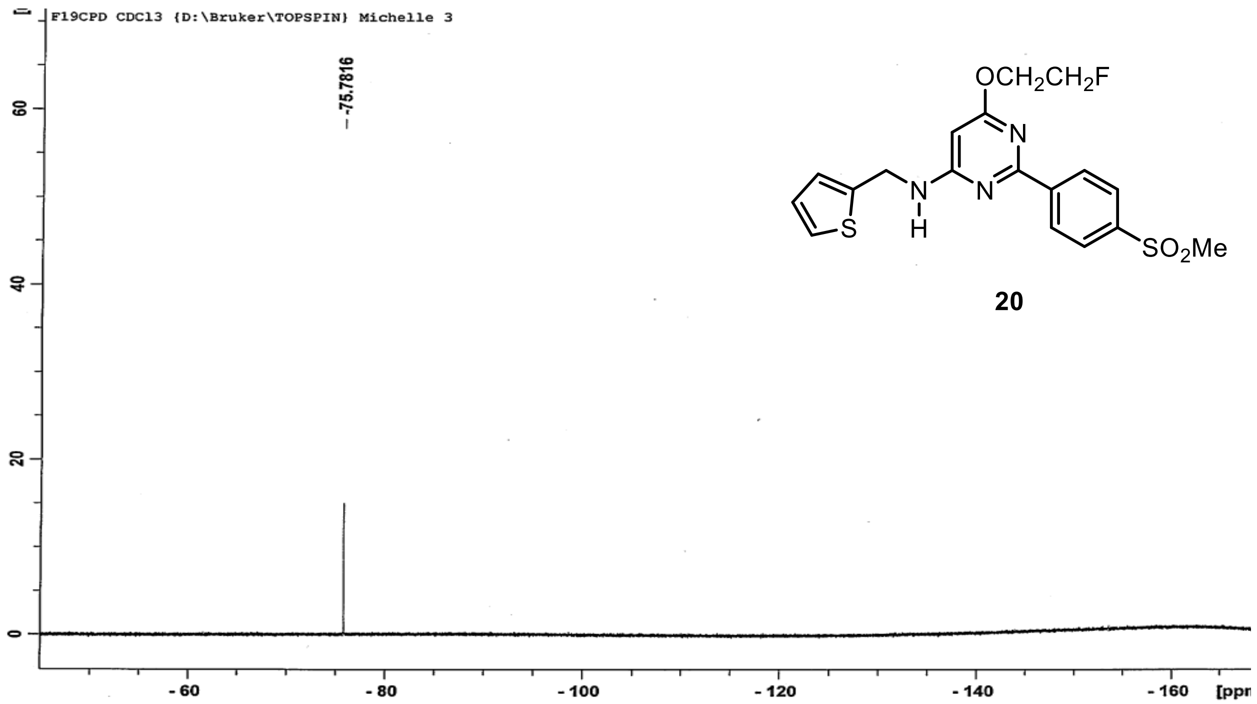
F19CPD CDCl3 {D:\Bruker\TOPSPIN} Michelle 3
-75.7816
OCH2CH2F
SO2Me
20
-60
-80
-100
-120
-140
-160
[ppm

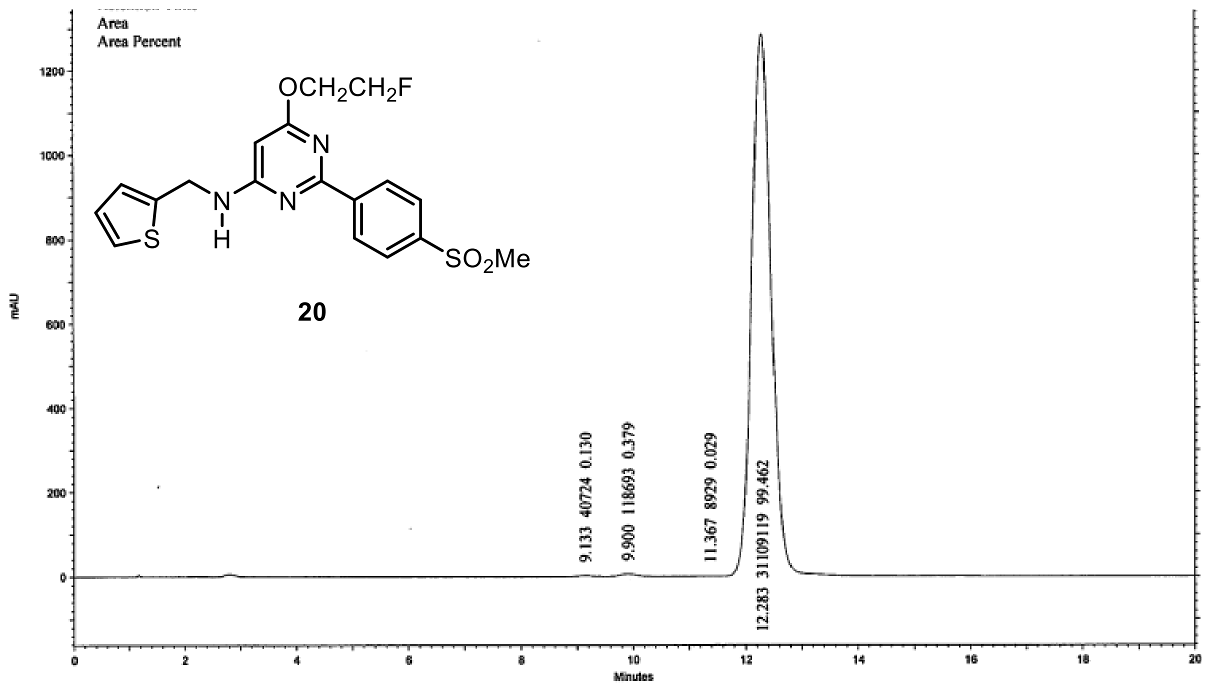
Area
Area Percent
OCH2CH2F
SO2Me
20
mAU
9.133 40724 0.130
9.900 118693 0.379
11.367 8929 0.029
12.283 31109119 99.462
Minutes

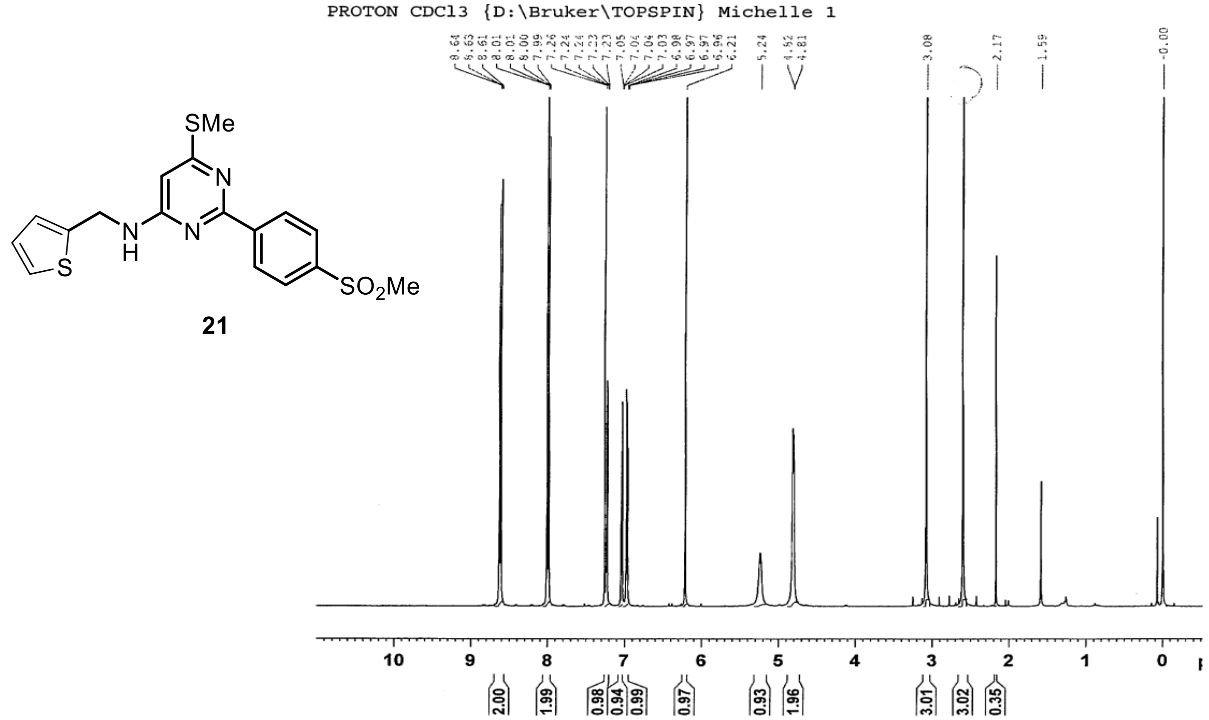
PROTON CDCl3 {D:\Bruker\TOPSPIN} Michelle 1

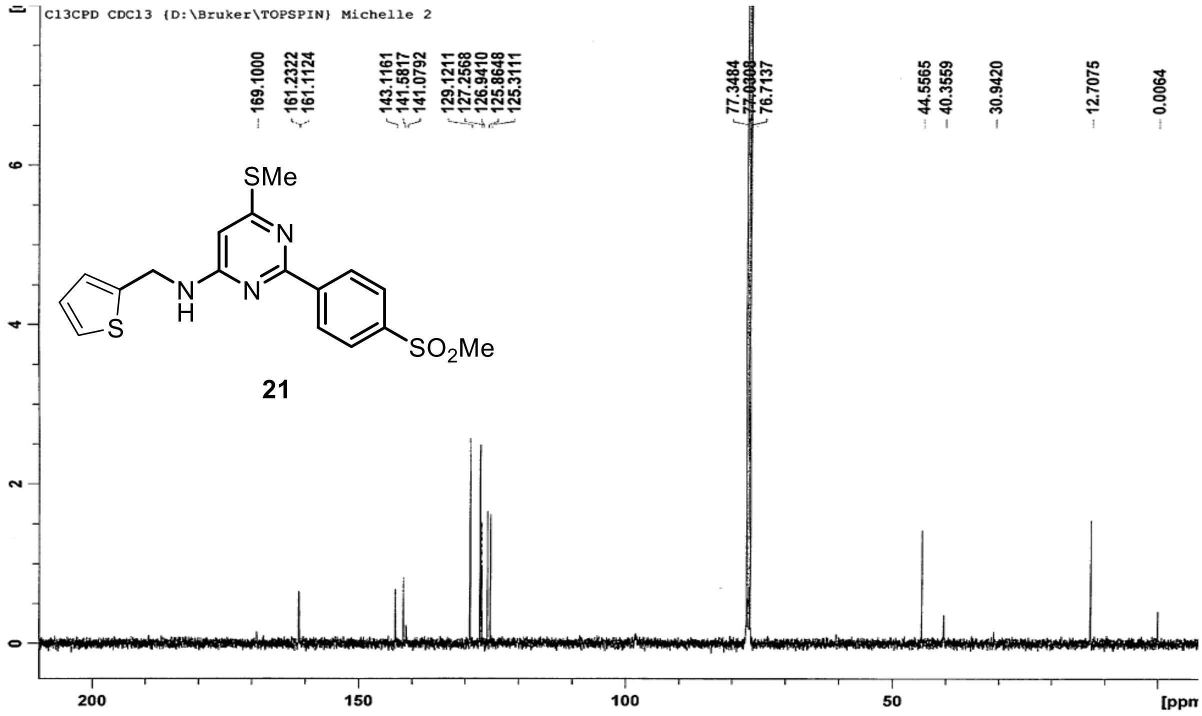
C13CPD CDCl3 {D:\Bruker\TOPSPIN} Michelle 2

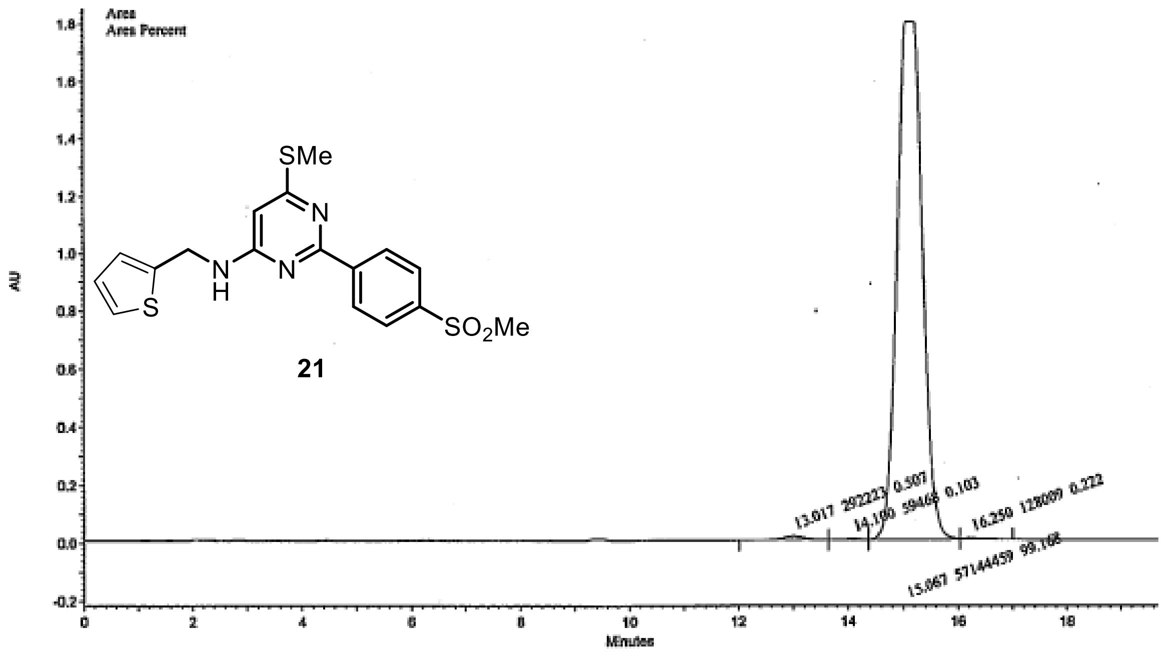
Area
Area Percent
SMe
N
N
H
N
S
SO2Me
21
AU
Minutes
13.017 292223 0.507
14.100 59468 0.103
16.250 128009 0.222
15.067 57144459 99.168

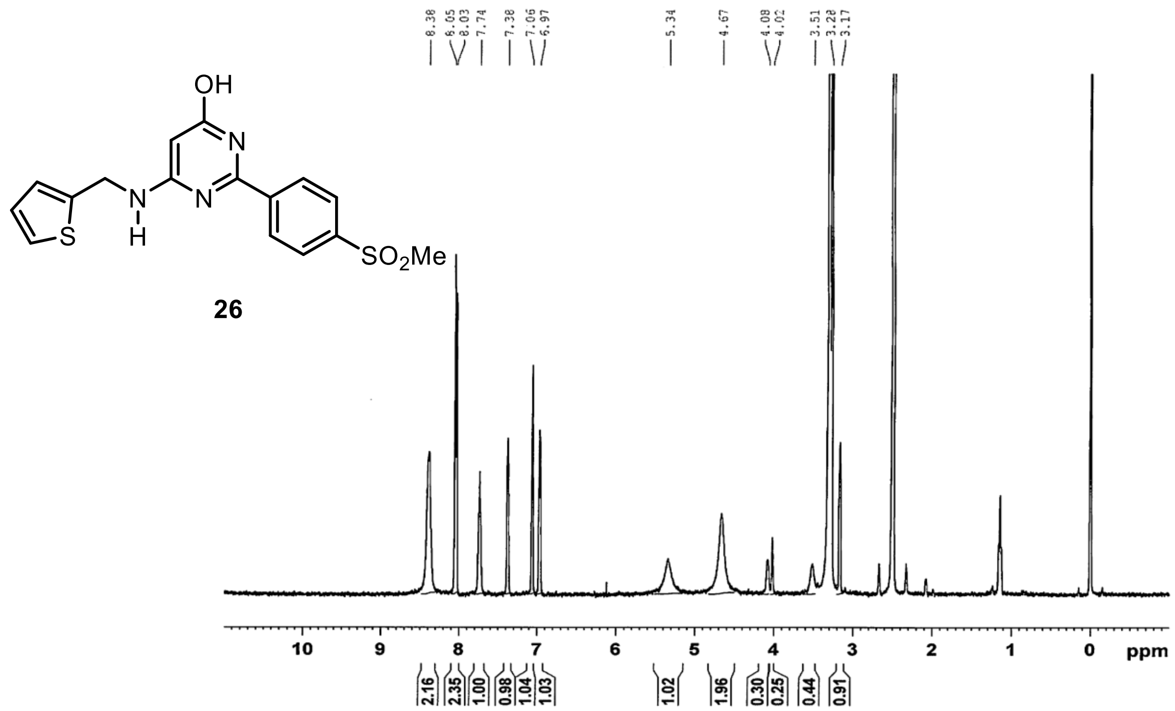
OH
N
N
N
H
S
SO2Me
8.38
8.05
8.03
7.74
7.38
7.06
6.97
5.34
4.67
4.08
4.02
3.51
3.17
10
9
8
7
6
5
4
3
2
1
0
ppm
2.16
2.35
1.00
0.98
1.04
1.03
1.02
1.96
0.30
0.25
0.44
0.91

26

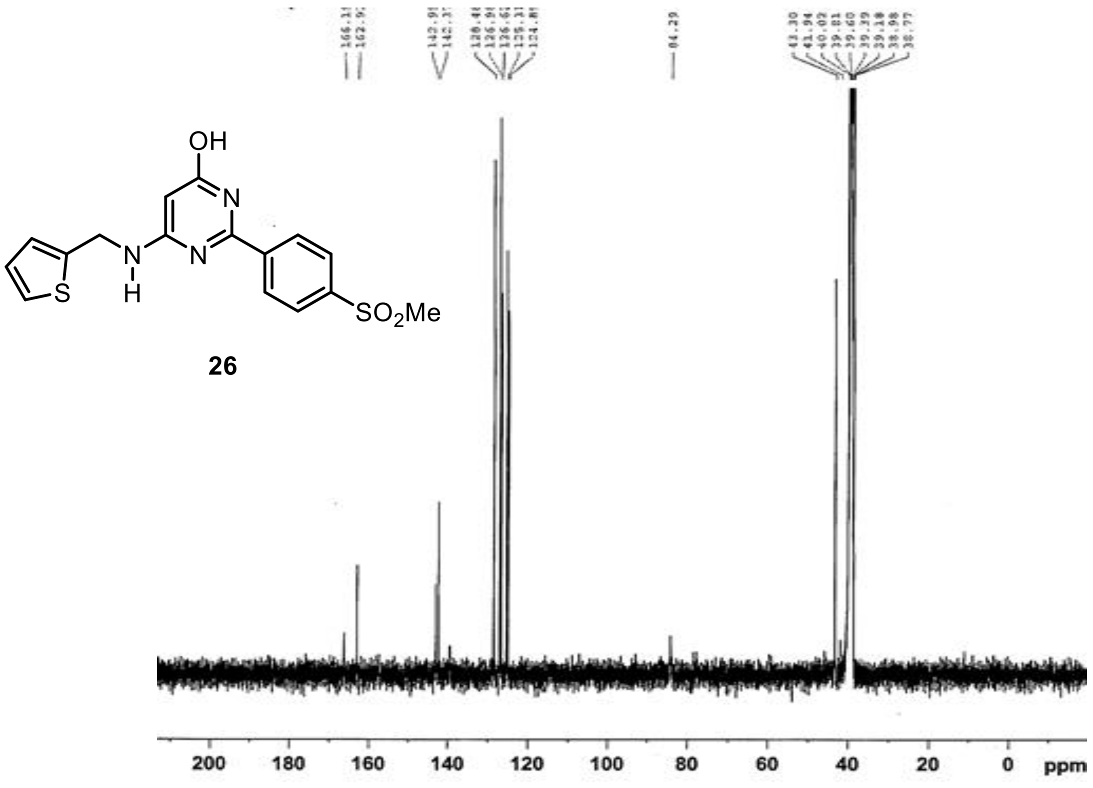
OH
N
N
N
H
S
SO2Me
84.29
200
180
160
140
120
100
80
60
40
20
0
ppm

26

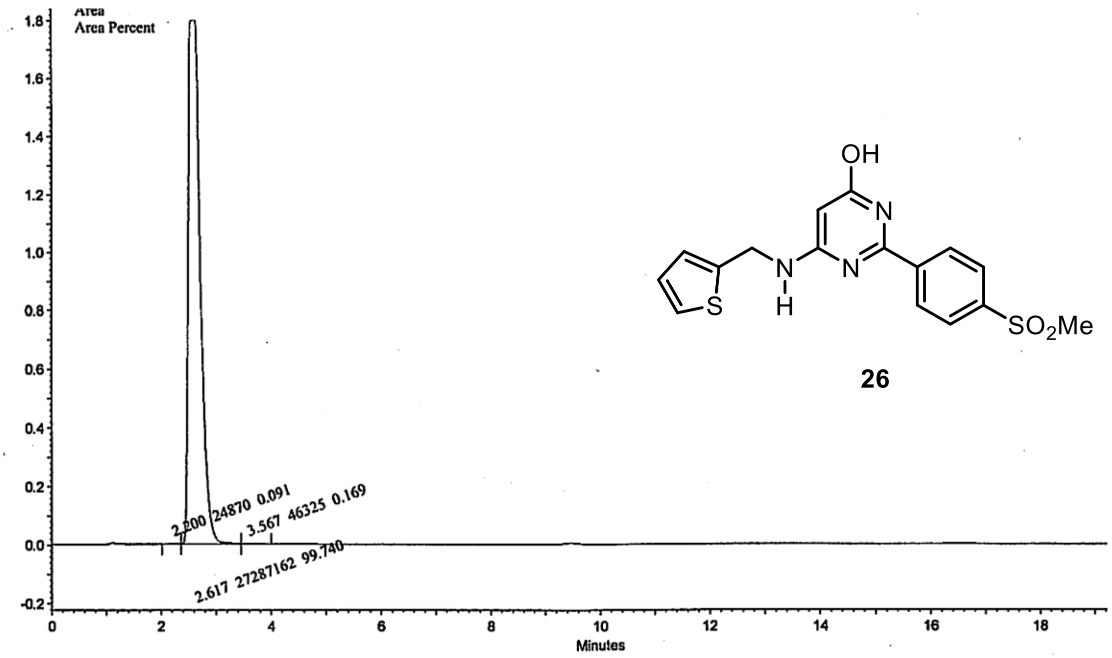
Area
Area Percent
2.200 24870 0.091
3.567 46325 0.169
2.617 27287162 99.740
Minutes
OH
N
S
H
SO2Me

26

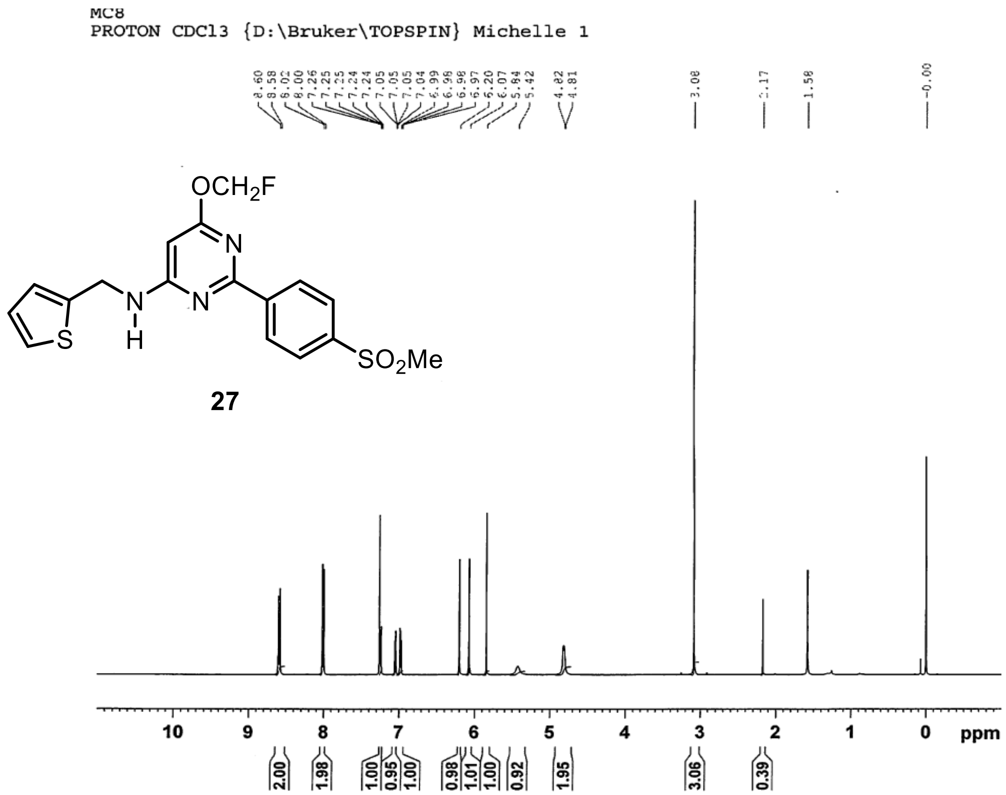
MC8
PROTON CDCl3 {D:\Bruker\TOPSPIN} Michelle 1
8.60 8.58 8.02 8.00 7.26 7.25 7.25 7.24 7.24 7.05 7.05 7.05 7.04 6.99 6.98 6.98 6.97 6.20 6.07 5.84 5.42 4.82 4.81 3.08 2.17 1.58 0.00
OCH2F
N
N
N
H
S
SO2Me
27
10 9 8 7 6 5 4 3 2 1 0 ppm
2.00 1.98 1.00 0.95 1.00 0.98 1.01 1.00 0.92 1.95 3.06 0.39

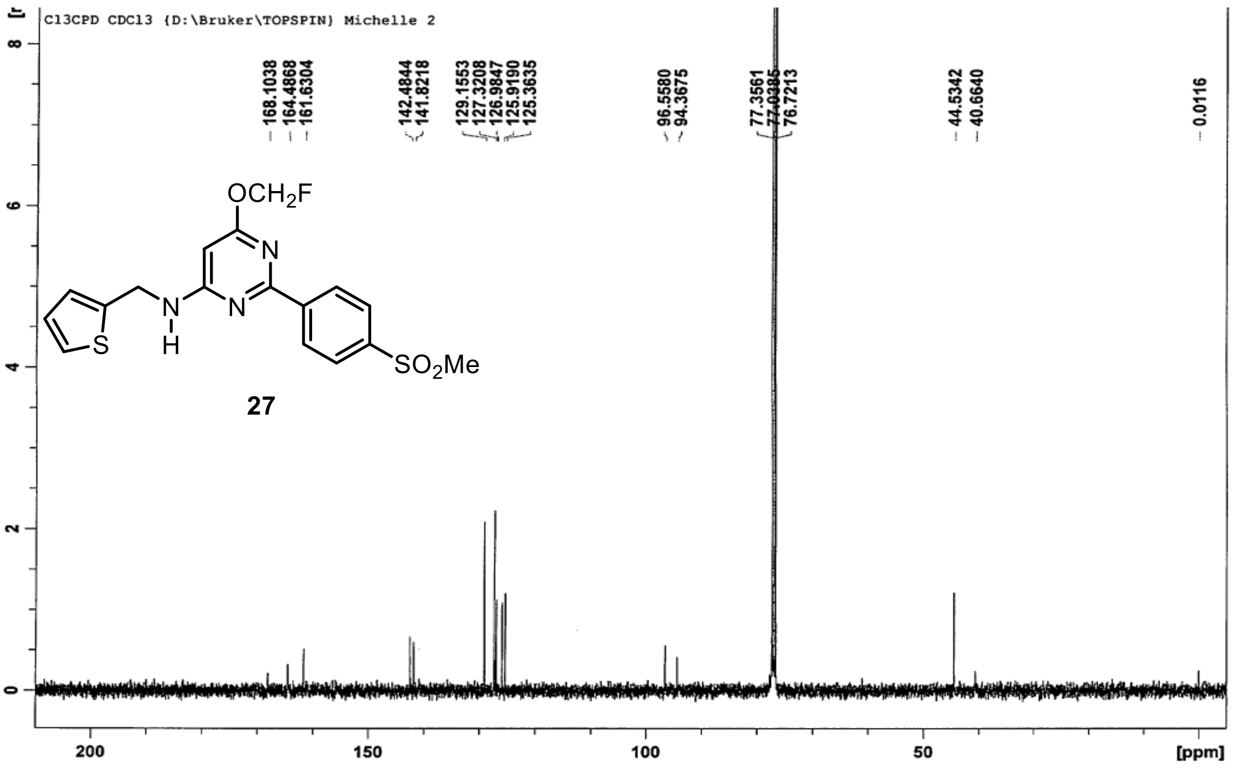
C13CPD CDCl3 {D:\Bruker\TOPSPIN} Michelle 2
168.1038 164.4868 161.6304 142.4844 141.8218 129.1553 127.3208 126.9847 125.9190 125.3635 96.5580 94.3675 77.3561 77.0385 76.7213 44.5342 40.6640 0.0116
OCH2F
N
N
N
H
S
SO2Me
27
8 6 4 2 0
200 150 100 50 [ppm]

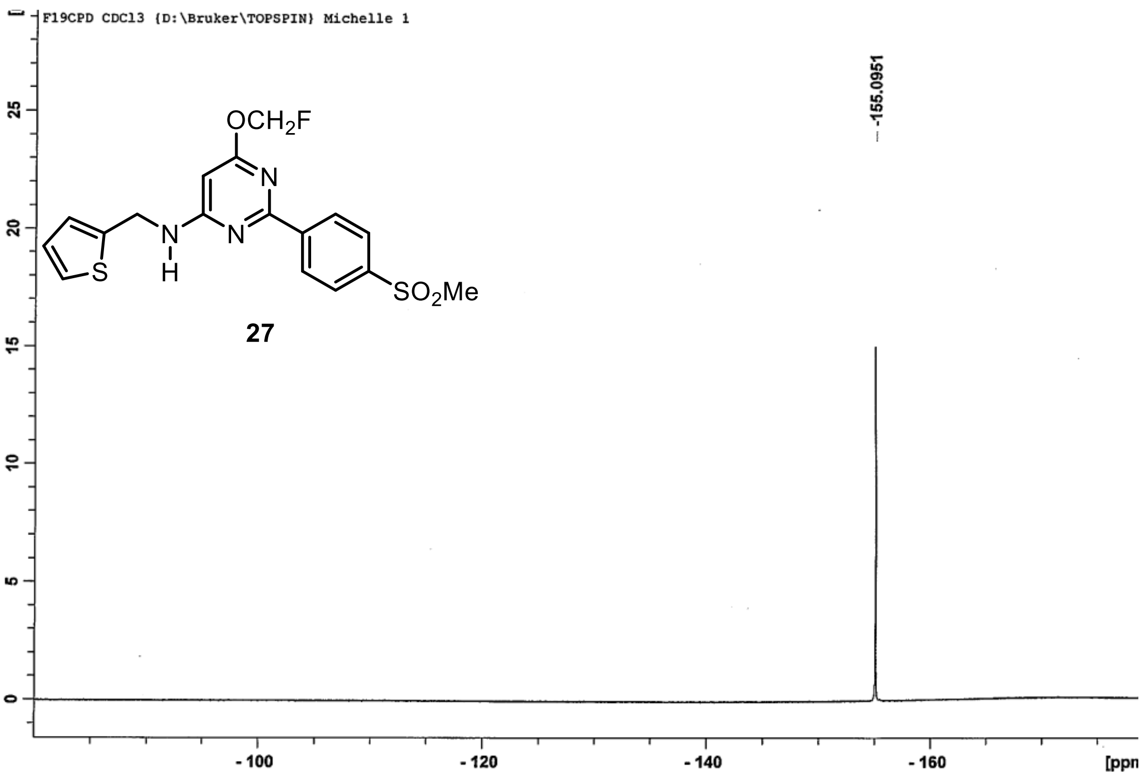
F19CPD CDCl3 {D:\Bruker\TOPSPIN} Michelle 1
-155.0951
OCH2F
N
N
N
H
S
SO2Me
27
- 100
- 120
- 140
- 160
[ppm

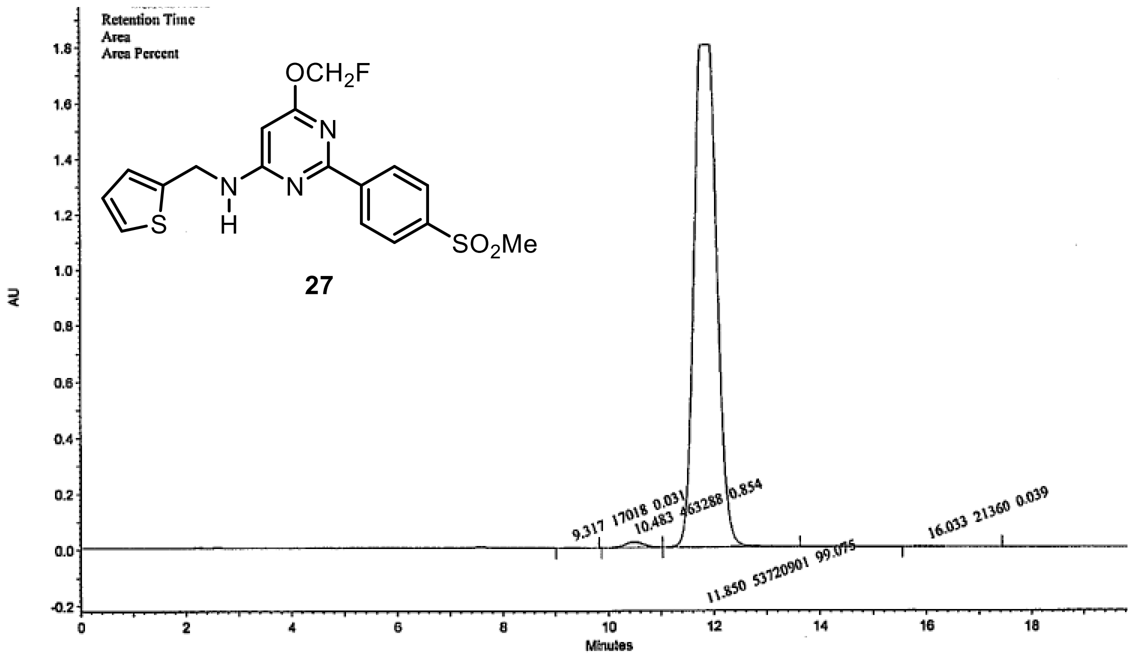
Retention Time
Area
Area Percent
OCH2F
N
N
N
H
S
SO2Me
27
AU
9.317 17018 0.031
10.483 463288 0.854
11.850 53720901 99.075
16.033 21360 0.039
Minutes

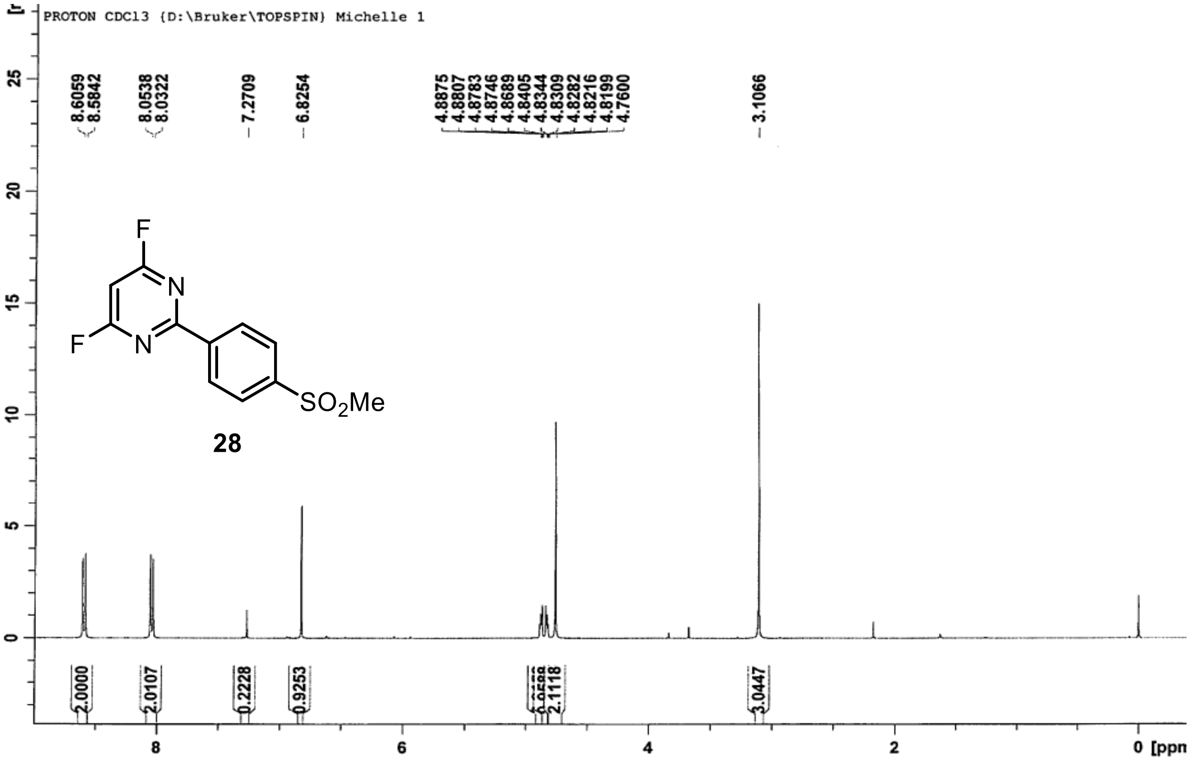
PROTON CDCl3 {D:\Bruker\TOPSPIN} Michelle 1
8.6059
8.5842
8.0538
8.0322
7.2709
6.8254
4.8875
4.8807
4.8783
4.8746
4.8689
4.8405
4.8344
4.8309
4.8282
4.8216
4.8199
4.7600
3.1066
2.0000
2.0107
0.2228
0.9253
2.1118
3.0447
F
N
F
N
SO2Me
28
[ppm]

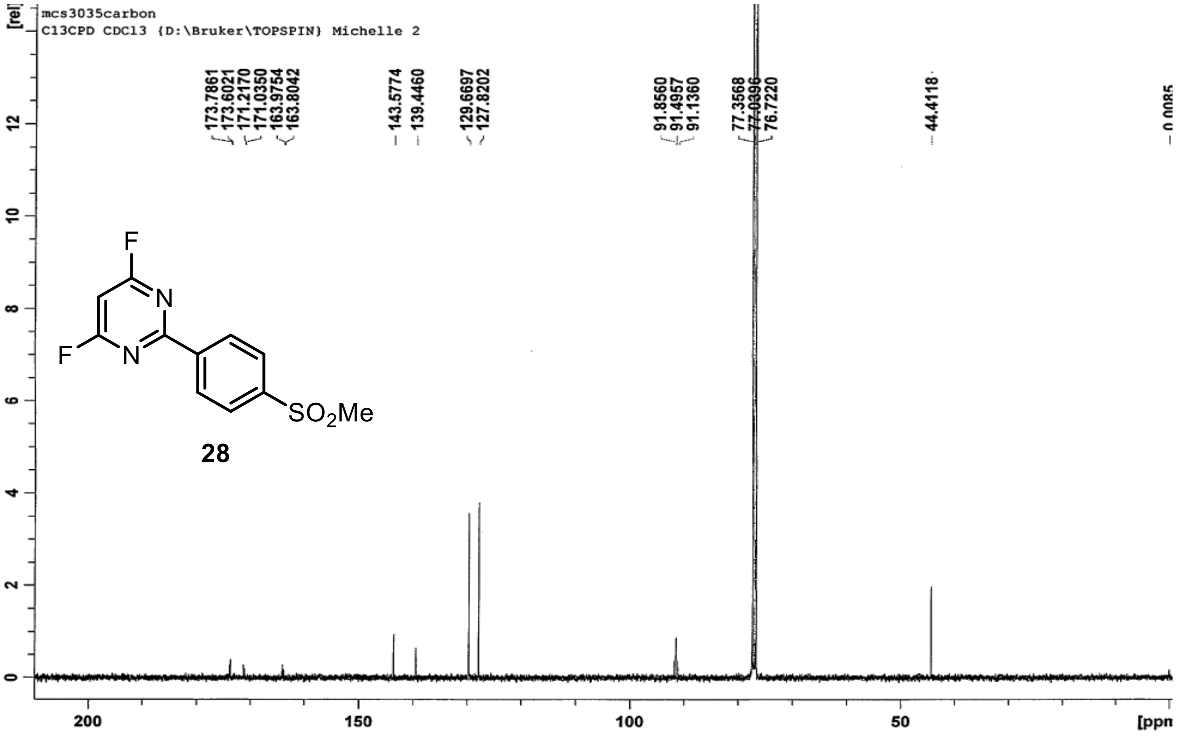
[rel]
mcs3035carbon
C13CPD CDCl3 {D:\Bruker\TOPSPIN} Michelle 2
173.7861
173.6021
171.2170
171.0350
163.9754
163.8042
143.5774
139.4460
129.6697
127.8202
91.8560
91.4957
91.1360
77.3568
76.7220
44.4118
F
N
F
N
SO2Me
28
[ppm]

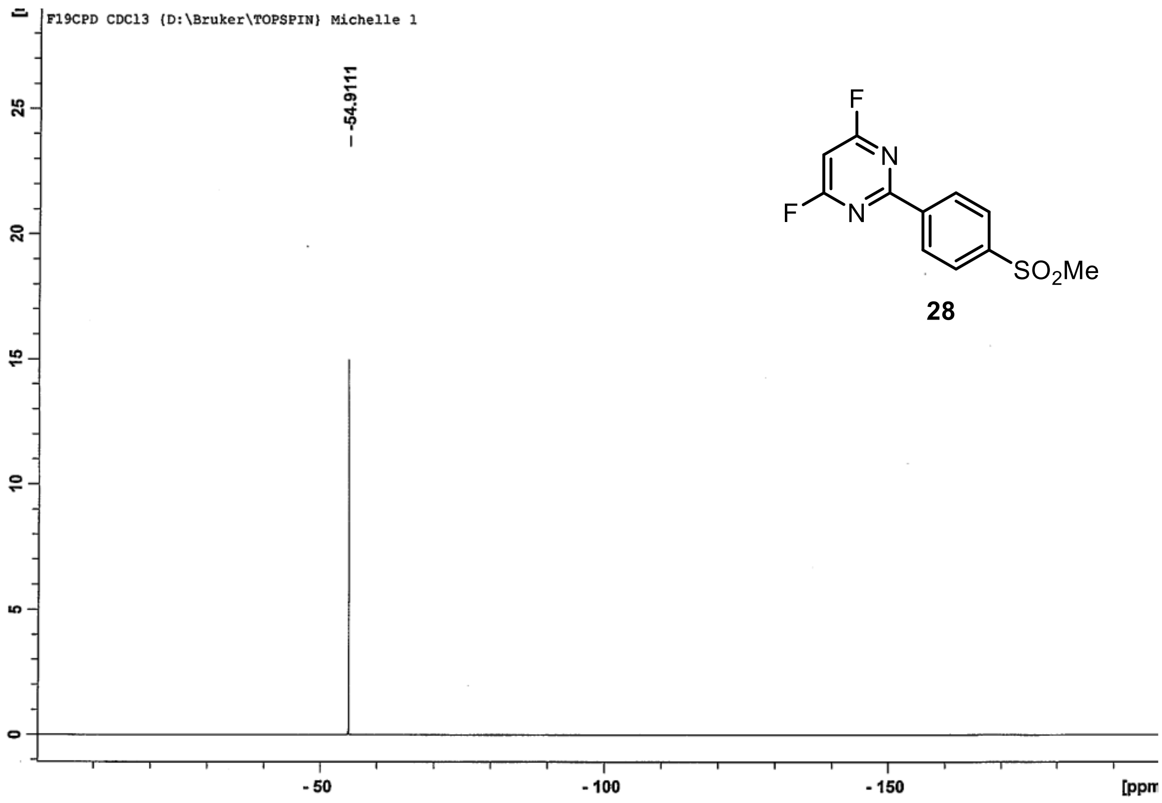
F19CPD CDCl3 {D:\Bruker\TOPSPIN} Michelle 1
-54.9111
F
N
F
N
SO2Me
28
25
20
15
10
5
0
-50
-100
-150
[ppm

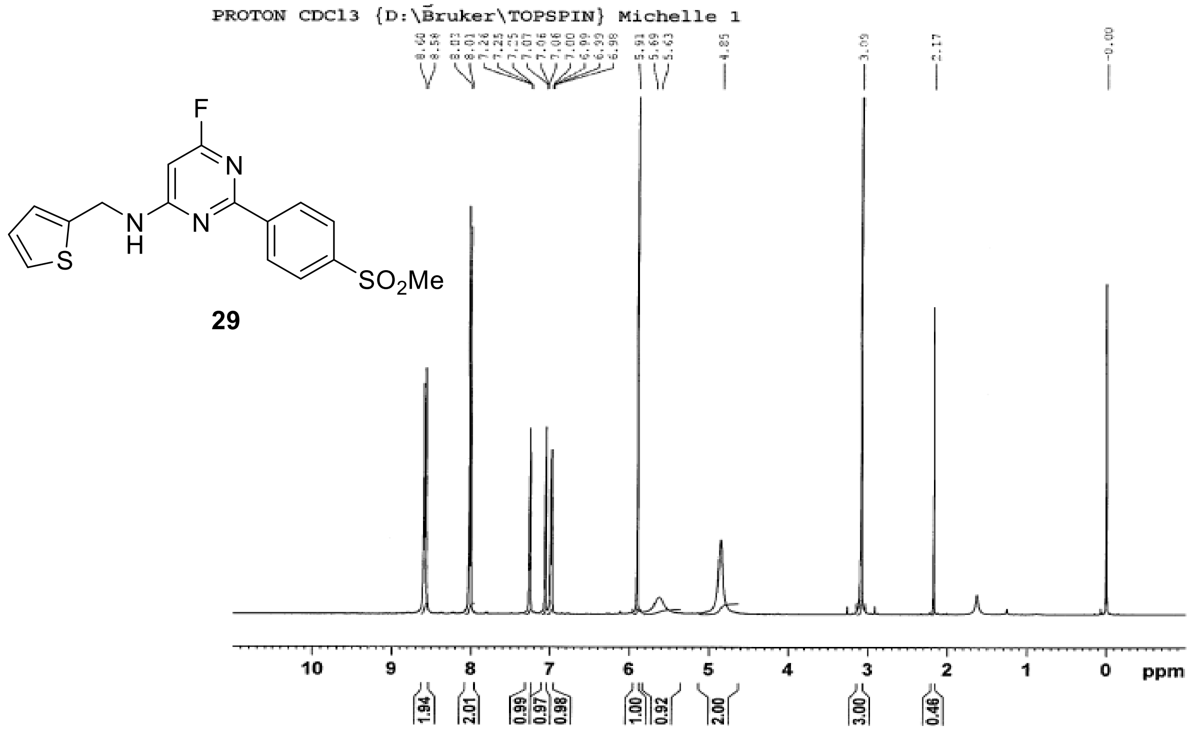
PROTON CDCl3 {D:\Bruker\TOPSPIN} Michelle 1
F
N
N
N
H
S
SO2Me
29
ppm
1.94
2.01
0.99
0.97
0.98
1.00
0.92
2.00
3.00
0.46

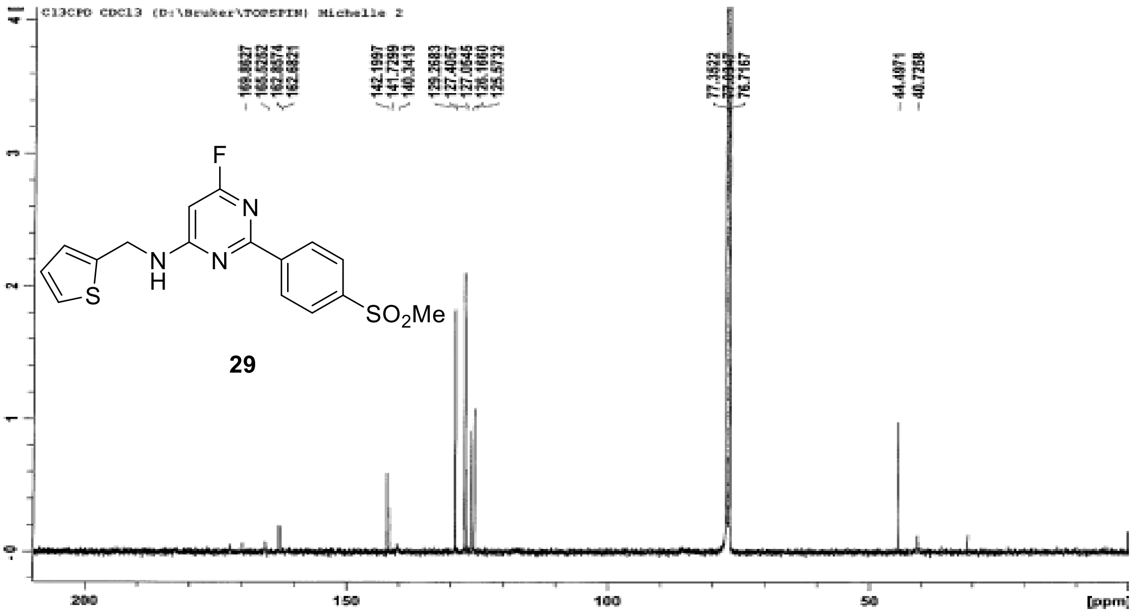
C13CPD CDCl3 {D:\Bruker\TOPSPIN} Michelle 2
F
N
N
N
H
S
SO2Me
29
[ppm]

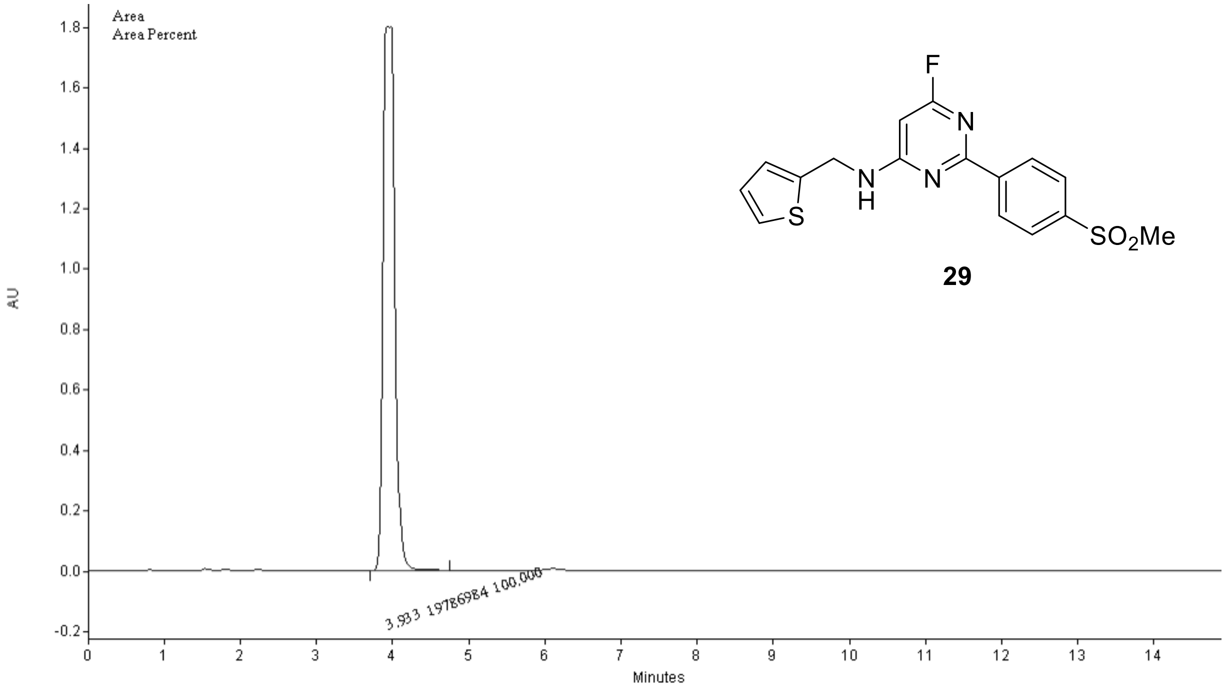
Area
Area Percent
AU
Minutes
3.933 19786984 100.000
F
N
N
NH
S
SO2Me
29

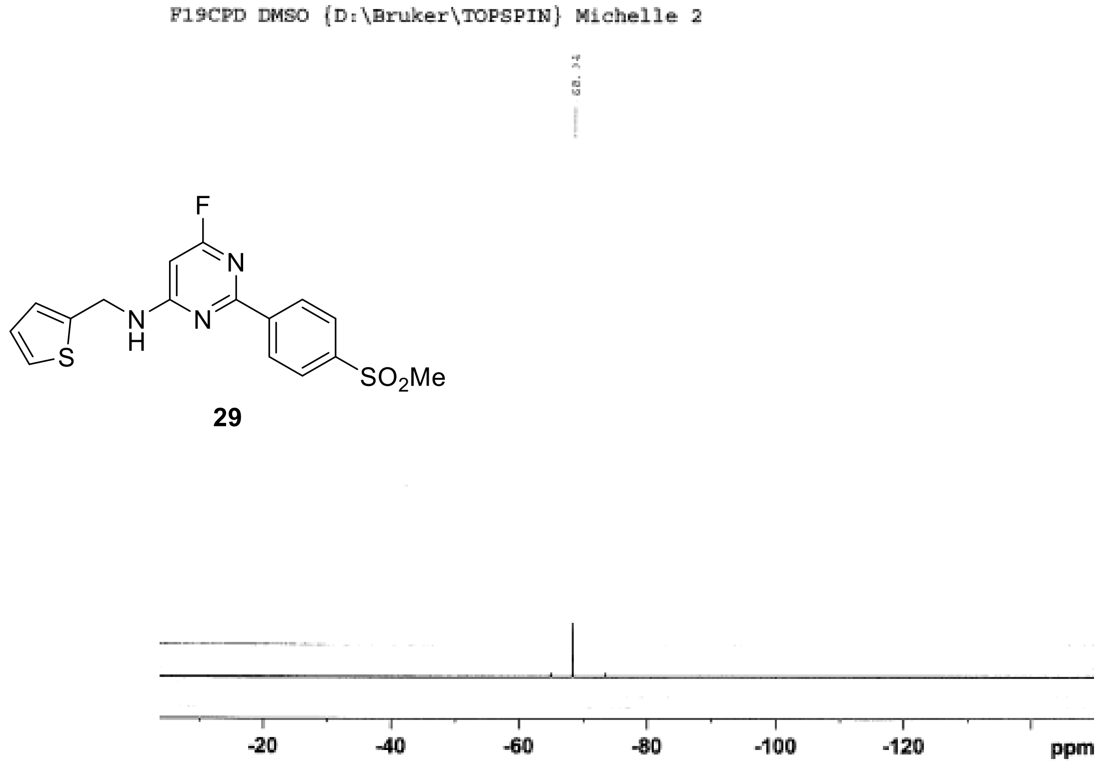
F19CPD DMSO {D:\Bruker\TOPSPIN} Michelle 2
F
N
N
NH
S
SO2Me
29
-20
-40
-60
-80
-100
-120
ppm

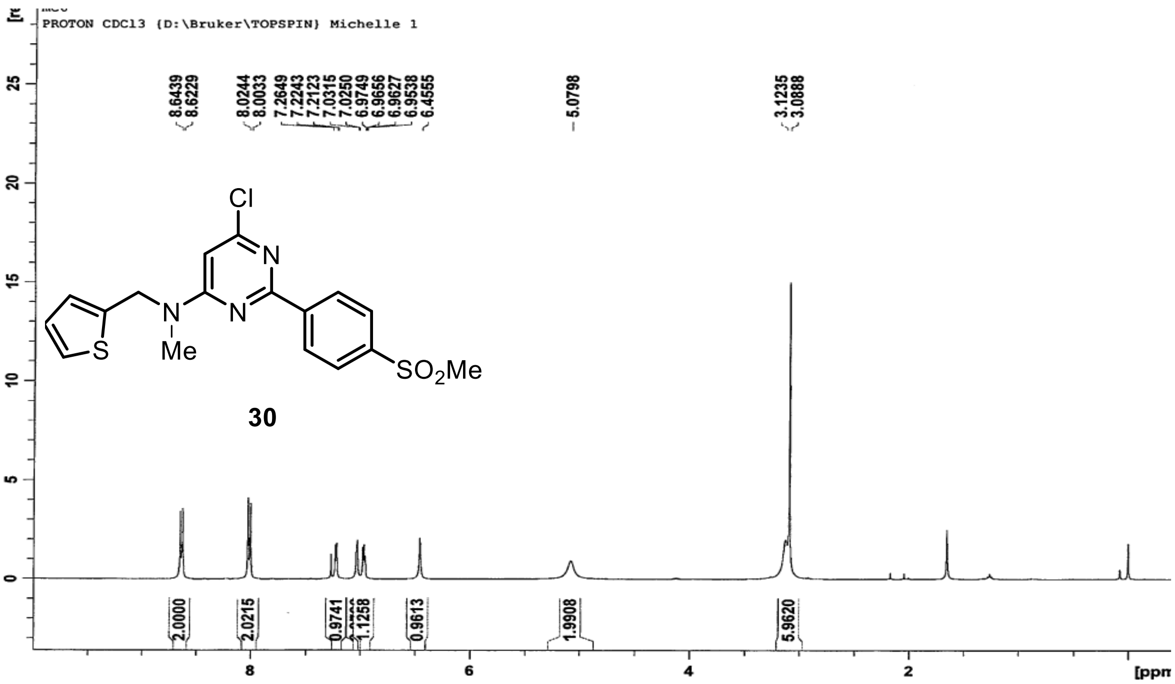
PROTON CDCl3 {D:\Bruker\TOPSPIN} Michelle 1
8.6439
8.6229
8.0244
8.0033
7.2649
7.2243
7.2123
7.0315
7.0250
6.9749
6.9656
6.9627
6.9538
6.4555
5.0798
3.1235
3.0888
Cl
N
N
N
S
Me
SO2Me
30
2.0000
2.0215
0.9741
1.1258
0.9613
1.9908
5.9620
[ppm]

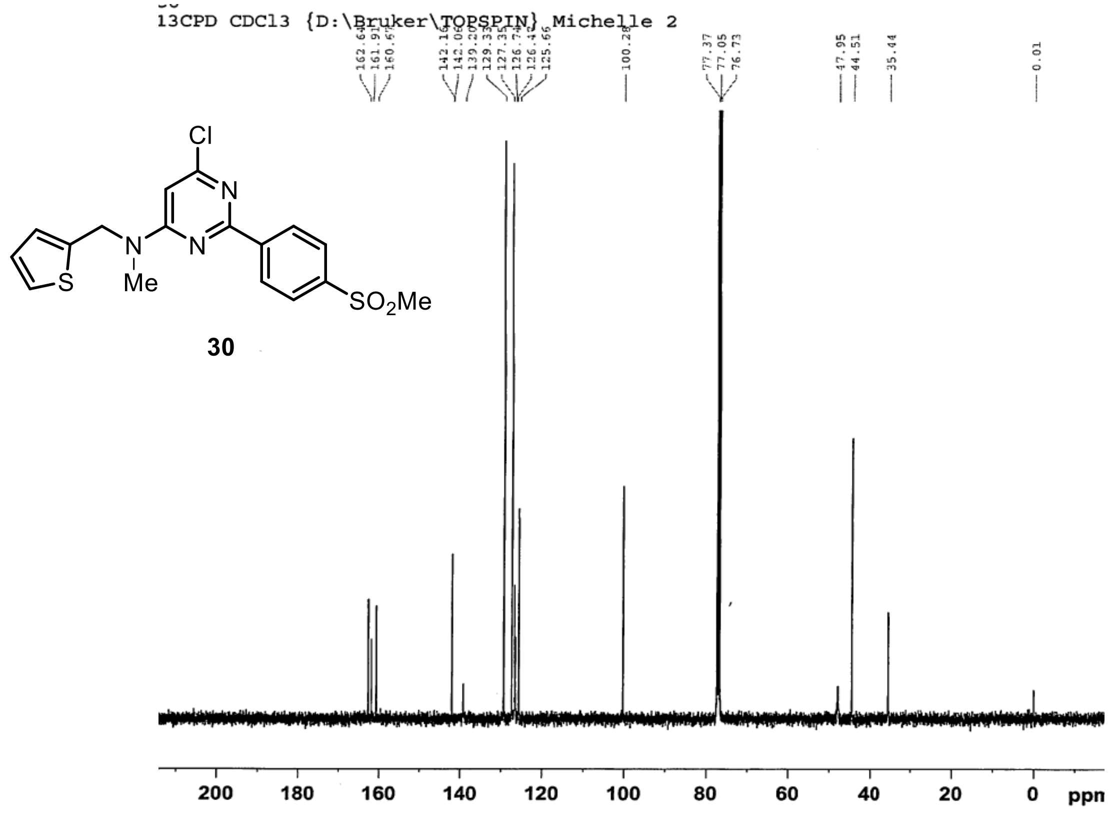
13CPD CDCl3 {D:\Bruker\TOPSPIN} Michelle 2
142.16
100.28
77.37
77.05
76.73
47.95
44.51
35.44
0.01
Cl
N
N
N
S
Me
SO2Me
30
ppm

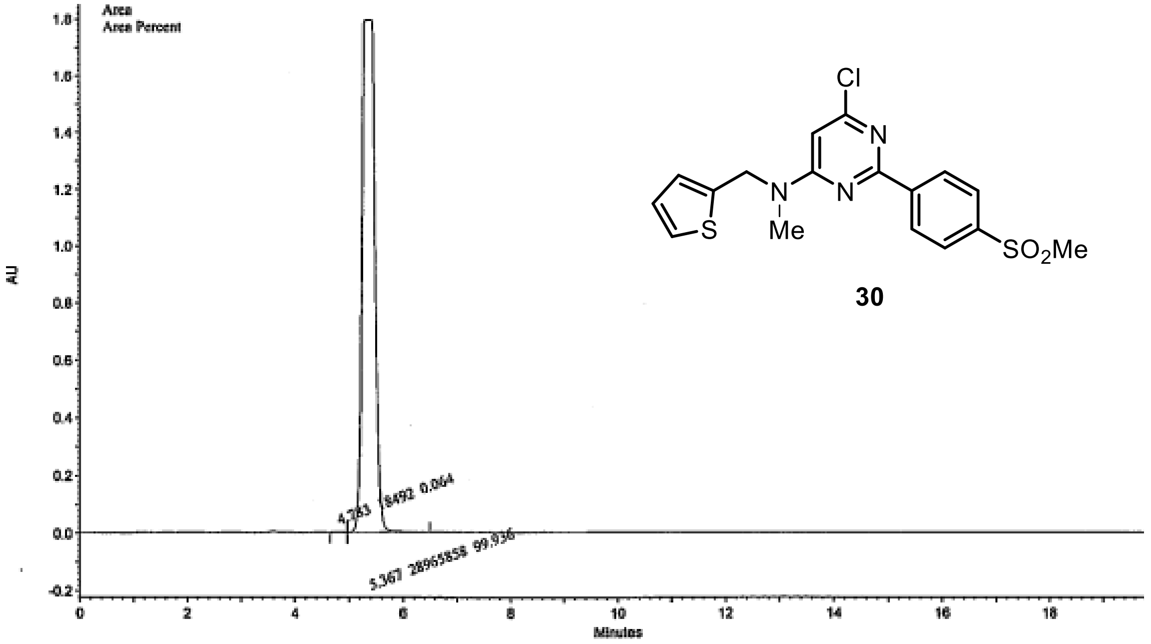

Area
Area Percent
AU
1.8
1.6
1.4
1.2
1.0
0.8
0.6
0.4
0.2
0.0
-0.2
0
2
4
6
8
10
12
14
16
18
Minutes
4.783
8492 0.064
5.367 28965858 99.936
Cl
N
N
N
Me
S
SO_2Me
30
